# Safe-RTSKF: A Safety-Constrained Recursive Two-Stage Kalman Filter for Robust Sensor State Estimation in Strongly Nonlinear Systems

**DOI:** 10.3390/s26123660

**Published:** 2026-06-08

**Authors:** Yifeng Lin, Ziyu Liu, Chenglin Wen, Ke Ye, Yang-kwon Jeong, Shiwei Zhou, Xuehong Zheng

**Affiliations:** 1 College of Automation, Guangdong University of Petrochemical Technology, Maoming 525000, China; linyifeng@gdupt.edu.cn (Y.L.); keye@gdupt.edu.cn (K.Y.); 2 Department of Computer Science, Dongshin University, Naju 58245, Republic of Korea; lzy717196@163.com (Z.L.); jovial@dsu.ac.kr (Y.-k.J.); 18772846185@163.com (S.Z.); zhengxuehong192@gmail.com (X.Z.)

**Keywords:** sensor state estimation, nonlinear filtering, Kalman filter, recursive two-stage Kalman filter, hidden variable consistency, robust filtering, outlier measurements, target tracking

## Abstract

Robust state estimation is essential for sensor systems operating under strong nonlinearity, high uncertainty, and outlier-contaminated measurements. Recursive two-stage Kalman filtering provides an interpretable framework for systems with multiplicative hidden variable decompositions, but its raw form may suffer from hidden variable drift and numerical instability when the hidden variable deviates from its structural relation with the state. To address this problem, this paper proposes Safe-RTSKF, a safety-constrained recursive two-stage Kalman filter for robust sensor state estimation. The proposed method integrates normalized innovation squared gating, spectral-norm Kalman gain clipping, update damping, Joseph-form covariance updating, covariance spectral projection, and hidden variable consistency correction. The consistency correction constrains the estimated hidden variable toward its structural relation with the updated state, thereby reducing recursive error accumulation. Theoretical analysis provides local boundedness and consistency error contraction conditions. Monte Carlo experiments on one-, two-, and four-dimensional strongly nonlinear systems show that Safe-RTSKF substantially improves the stability of Raw RTSKF. Additional range–bearing and coordinated turn tracking experiments demonstrate that the safety update mechanism can also improve EKF robustness under outlier measurements. The results indicate that the proposed framework offers a practical accuracy–stability–runtime trade-off for nonlinear sensor state estimation.

## 1. Introduction

Robust state estimation is a fundamental task in modern sensor systems, including target tracking, navigation, mobile robotics, industrial monitoring, fault diagnosis, and autonomous sensing. In these applications, the internal system state is usually not directly observable and must be recursively inferred from noisy sensor measurements. Kalman filtering provides a classical recursive estimation framework for linear Gaussian systems and has been widely extended to nonlinear sensor state estimation problems because of its relatively low computational cost and clear probabilistic interpretation [1,2].

However, practical sensor state estimation often involves strong nonlinearity, high initial uncertainty, model mismatch, and outlier-contaminated measurements. For example, range–bearing sensors introduce nonlinear measurement functions, maneuvering targets exhibit nonlinear motion dynamics, and industrial sensing systems may contain multiplicative coupling terms between latent states and structural variables. Under these conditions, conventional nonlinear Kalman filters may suffer from degraded accuracy or numerical instability. The extended Kalman filter (EKF) relies on local linearization and may be sensitive to large linearization errors. The unscented Kalman filter (UKF), Gaussian filters, and cubature Kalman filter (CKF) improve nonlinear propagation through deterministic sampling, but their performance may still deteriorate under strong local nonlinearity, high uncertainty, or abnormal innovations [3,4,5,6]. Particle filters (PFs) provide a more general Bayesian approximation for nonlinear and non-Gaussian systems, but their computational cost increases rapidly with the number of particles and the state dimension [7,8].

Robust and adaptive filtering methods have been developed to reduce the influence of abnormal measurements, unknown noise statistics, and non-Gaussian disturbances. Representative approaches include robust Bayesian filtering, Huber-type robust estimation, innovation-based adaptive covariance estimation, $H_{\infty }$ filtering, high-degree cubature filtering, robust iterated EKF, and correntropy-based nonlinear filtering [9,10,11,12,13,14,15,16,17]. These methods improve the robustness of recursive estimation from different perspectives, but many of them focus mainly on measurement-update robustness rather than on the structural consistency between state-dependent hidden variables and the main state estimate.

Recursive two-stage Kalman filtering (RTSKF) provides a structured way to estimate systems in which the original nonlinear dynamics can be decomposed into a state variable and a hidden variable. Related two-stage or separate-bias filtering ideas were originally developed for recursive estimation with unknown bias, random bias, or exogenous inputs [18,19,20,21,22,23]. This structure is useful for strongly nonlinear systems with multiplicative hidden variable relationships. Instead of directly filtering a high-dimensional augmented state, RTSKF separately updates the main state and the hidden variable, thereby improving interpretability and retaining a recursive Kalman-type implementation. Nevertheless, the raw RTSKF structure may suffer from hidden variable drift. When the estimated hidden variable gradually deviates from its structural relation with the estimated state, the prediction model receives a persistent biased input. This error may be recursively amplified and may eventually lead to covariance inflation, unstable updates, or filtering divergence.

Kalman-type filters have also been widely used in sensor applications, including maneuvering target tracking, underwater navigation, GPS positioning, state-of-charge estimation, high-speed train fault detection, nonlinear non-Gaussian filtering, state-saturated systems, high-order Kalman filtering, multisensor estimation, pedestrian tracking, and transportation monitoring [24,25,26,27,28,29,30,31,32,33,34,35,36,37,38]. These applications show that recursive filtering remains attractive for sensor systems because of its online implementation capability. However, abnormal innovations, strong maneuvering dynamics, and hidden structural variables still pose challenges to stable and reliable recursive estimation.

To address this problem, this paper proposes Safe-RTSKF, a safety-constrained recursive two-stage Kalman filter for robust sensor state estimation in strongly nonlinear systems. The key idea is to enhance the raw two-stage recursive filtering process from both numerical and structural perspectives. Specifically, normalized innovation squared (NIS) gating is used to suppress abnormal innovations; spectral-norm Kalman gain clipping and update damping are introduced to limit excessive correction steps; Joseph-form covariance updating and covariance spectral projection are used to maintain covariance numerical stability; and a hidden variable consistency correction is designed to constrain the updated hidden variable toward its structural relation with the estimated state. In this way, the proposed method not only limits unsafe recursive updates but also reduces hidden variable inconsistency, which is a specific instability source in RTSKF-type models.

The proposed method is not intended to replace all nonlinear Bayesian filters or to claim universal superiority over EKF, UKF, CKF, or PF. Instead, it is positioned as a practical stability enhancement framework for recursive sensor state estimation under strong nonlinearity and outlier-contaminated measurements. In addition to evaluating Safe-RTSKF on strongly nonlinear hidden variable systems, this study further transfers the safety update mechanism to the EKF framework and constructs a Safe-EKF for range–bearing and coordinated turn tracking scenarios. These experiments are used to examine whether the proposed safety update idea can improve robustness in sensor tracking problems involving abnormal measurements and maneuvering dynamics.

The main contributions of this study are summarized as follows:

1. A safety-constrained recursive two-stage Kalman filtering framework is proposed for strongly nonlinear sensor state estimation problems with multiplicative hidden variable structures.
2. A hidden variable consistency correction mechanism is introduced to constrain the updated hidden variable toward its structural relation with the estimated state, thereby reducing recursive drift in Raw RTSKF.
3. Safety update mechanisms, including NIS gating, spectral-norm gain clipping, update damping, Joseph-form covariance updating, and covariance spectral projection, are systematically embedded into the two-stage recursive filtering process.
4. Local theoretical analysis is provided to explain the boundedness of the safety-constrained update and the contraction condition of the hidden variable consistency error.
5. Extensive Monte Carlo experiments, ablation studies, parameter sensitivity analysis, runtime comparison, range–bearing tracking, and coordinated turn benchmark experiments are conducted to evaluate the accuracy, robustness, and computational efficiency of the proposed framework.

The remainder of this paper is organized as follows. Section 2 presents the problem formulation, the proposed Safe-RTSKF method, the theoretical analysis, and the Safe-EKF extension for range–bearing sensor tracking. Section 3 reports the experimental settings and results. Section 4 discusses the findings, limitations, and practical implications. Section 5 concludes the paper.

## 2. Materials and Methods

### 2.1. Problem Formulation for Nonlinear Sensor State Estimation

Consider a discrete-time nonlinear sensor state estimation problem described by the following state space model, which is commonly used in recursive Kalman-type sensor estimation frameworks [1,2,5,6]

(1) $$x_{k+1}=F\left(x_{k}\right)+w_{k},$$

(2) $$z_{k+1}=H\left(x_{k+1}\right)+\varepsilon_{k+1},$$

where $x_{k}\in R^{n}$ denotes the latent system state, $z_{k}\in R^{m}$ denotes the sensor measurement, and $w_{k}$ and $\varepsilon_{k}$ are the process and measurement noise terms, respectively. In many sensor systems, the nonlinear dynamics or measurement functions may contain multiplicative or structurally coupled terms. Directly filtering such systems may lead to large approximation errors or unstable recursive updates, especially under high uncertainty or outlier-contaminated measurements.

To avoid ambiguity, the notation used throughout the revised manuscript is summarized in Table 1. In particular, $z_{k}$ is consistently used for measurements, $\varepsilon_{k}$ for measurement noise, and $v_{k}$ is reserved only for the target speed in the coordinated turn benchmark.

For strongly nonlinear systems with a multiplicative hidden variable structure, this study introduces a hidden variable

(3) $$\alpha_{k}=f\left(x_{k}\right),$$

and rewrites the state transition model as

(4) $$x_{k+1}=G\left(x_{k},\alpha_{k}\right)+w_{k}.$$

The corresponding sensor measurement model is written as

(5) $$z_{k+1}=M\left(x_{k+1},\alpha_{k+1}\right)+\varepsilon_{k+1}.$$

Here, $\alpha_{k}$ is not an independent external bias but a structural hidden variable determined by the system state. Therefore, a reliable recursive filtering algorithm should estimate both $x_{k}$ and $\alpha_{k}$ while maintaining their structural consistency.

To illustrate this type of system, the one-dimensional nonlinear model used in this study is

(6) $$x_{k+1}=x_{k}\sin\left(\beta x_{k}\right)+w_{k},$$

(7) $$z_{k+1}=\sin\left(x_{k+1}\right)\cos\left(\sin\left(\beta x_{k+1}\right)\right)+\varepsilon_{k+1}.$$

By defining

(8) $$\alpha_{k}=\sin\left(\beta x_{k}\right),$$

the state transition can be rewritten as

(9) $$x_{k+1}=x_{k}\alpha_{k}+w_{k}.$$

A two-dimensional coupled model is also considered

(10) $$x_{1,k+1}=0.8x_{1,k}\cos\left(\beta_{1}x_{2,k}\right)+w_{1,k},$$

(11) $$x_{2,k+1}=0.8x_{2,k}\cos\left(\beta_{2}x_{1,k}\right)+w_{2,k},$$

with hidden variables

(12) $$\alpha_{1,k}=\cos\left(\beta_{1}x_{2,k}\right),\;\alpha_{2,k}=\cos\left(\beta_{2}x_{1,k}\right).$$

These examples represent nonlinear sensor estimation problems in which the hidden variables are structurally coupled with the state rather than being independent random biases.

### 2.2. Recursive Two-Stage Kalman Filtering and Hidden Variable Drift

Recursive two-stage Kalman filtering estimates the main state and the hidden variable in a structured recursive manner. This idea is related to separate-bias and two-stage Kalman estimation, which were originally developed to handle unknown or random bias without directly constructing a full augmented-state filter [18,19,20,21,22,23]. Compared with a direct augmented-state filter, the two-stage structure can improve interpretability and reduce the difficulty of directly approximating a strongly nonlinear mapping. In the raw RTSKF formulation, the state prediction is performed using the current state and hidden variable estimates

(13) $${\hat{x}}_{k+1|k}=G\left({\hat{x}}_{k|k},{\hat{\alpha }}_{k|k}\right).$$

The hidden variable is then predicted and updated according to the sensor measurement. However, because the hidden variable is estimated recursively, its estimate may gradually deviate from its structural relation with the state

(14) $${\hat{\alpha }}_{k|k}\neq f\left({\hat{x}}_{k|k}\right).$$

This inconsistency is referred to as hidden variable drift in this paper.

Hidden variable drift is a critical instability source for multiplicative nonlinear systems. Once ${\hat{\alpha }}_{k|k}$ deviates from $f({\hat{x}}_{k|k})$, the state prediction in Equation (13) receives a biased structural input. The resulting prediction error may further affect the subsequent measurement update and hidden variable update, leading to recursive error accumulation. Under high initial uncertainty, strong local nonlinearity, or outlier-contaminated sensor measurements, this process may cause covariance inflation, abnormal correction steps, or filtering divergence. Therefore, improving Raw RTSKF requires not only robust measurement updates but also a mechanism for maintaining the consistency between the hidden variable and the state.

### 2.3. Proposed Safe-RTSKF

To address the hidden variable drift and numerical instability problems, this paper proposes Safe-RTSKF, a safety-constrained recursive two-stage Kalman filter. The proposed method enhances Raw RTSKF from two complementary perspectives. First, unsafe recursive updates are limited by normalized innovation squared (NIS) gating, spectral-norm Kalman gain clipping, update damping, Joseph-form covariance updating, and covariance spectral projection. Second, the hidden variable estimate is corrected using the structural relation α=f(x), thereby reducing the recursive accumulation of hidden-variable inconsistency.

#### 2.3.1. State Prediction

Given the posterior estimates ${\hat{x}}_{k|k}$ and ${\hat{\alpha }}_{k|k}$, the state prediction is computed as

(15) $${\hat{x}}_{k+1|k}=G\left({\hat{x}}_{k|k},{\hat{\alpha }}_{k|k}\right).$$

Let

(16) $$G_{x}={\left.\frac{\partial G(x,\alpha )}{\partial x}\right|}_{{\hat{x}}_{k|k},{\hat{\alpha }}_{k|k}},\;G_{\alpha }={\left.\frac{\partial G(x,\alpha )}{\partial \alpha }\right|}_{{\hat{x}}_{k|k},{\hat{\alpha }}_{k|k}}.$$

The state prediction covariance is approximated by

(17) $$P_{k+1|k}^{x}=G_{x}P_{k|k}^{x}G_{x}^{T}+G_{\alpha }P_{k|k}^{\alpha }G_{\alpha }^{T}+Q_{k}+\Delta G_{k},$$

where $P_{k|k}^{x}$ and $P_{k|k}^{\alpha }$ are the state and hidden variable covariance matrices, respectively; $Q_{k}$ is the process noise covariance; and $\Delta G_{k}$ denotes a compensation term for multiplicative approximation errors. For the one-dimensional multiplicative model $x_{k+1}=x_{k}\alpha_{k}+w_{k}$, Equation (17) reduces to

(18) $$P_{k+1|k}^{x}\approx {\hat{\alpha }}_{k|k}^{2}P_{k|k}^{x}+{\hat{x}}_{k|k}^{2}P_{k|k}^{\alpha }+P_{k|k}^{x}P_{k|k}^{\alpha }+Q_{k}.$$

The term $P_{k|k}^{\alpha }$ in Equation (17) is chosen to approximate the local uncertainty of the nonlinear structural term $f(x_{k})$. Since $\alpha_{k}=f\left(x_{k}\right)$ is state-dependent, a first-order uncertainty propagation gives

(19) $$P_{k|k}^{\alpha }\approx J_{f,k}P_{k|k}^{x}J_{f,k}^{T}+Q_{k}^{\alpha ,\mathrm{str}},$$

where $J_{f,k}{=\partial f\left(x\right)/\partial x|}_{{\hat{x}}_{k|k}}$, and $Q_{k}^{\alpha ,\mathrm{str}}$ is a small positive semidefinite inflation term accounting for higher-order linearization error and possible structural mismatch. In the implementation, $P_{k|k}^{\alpha }$ is also projected to $\left[P_{\min},P_{\alpha ,\max}\right]$ to prevent collapse or excessive inflation.

The compensation term $\Delta G_{k}$ in Equation (17) is introduced because $G(x_{k},\alpha_{k})$ is multiplicative or structurally coupled. For the scalar product $x_{k}\alpha_{k}$, if $x_{k}$ and $\alpha_{k}$ are locally unbiased and approximately independent after conditioning, the variance contains the additional term

(20) $$\mathrm{Var}\left(x_{k}\alpha_{k}\right)\approx {\hat{\alpha }}_{k|k}^{2}P_{k|k}^{x}+{\hat{x}}_{k|k}^{2}P_{k|k}^{\alpha }+P_{k|k}^{x}P_{k|k}^{\alpha }.$$

Thus, $\Delta G_{k}$ is not an arbitrary tuning term; it represents residual multiplicative uncertainty not captured by the two linear terms. In multidimensional experiments, it is chosen as a small positive semidefinite inflation,

(21) $$\Delta G_{k}=\eta_{G}\;\mathrm{diag}\;\left(G_{\alpha }P_{k|k}^{\alpha }G_{\alpha }^{T}\right),\;\eta_{G}\geq 0,$$

and $\eta_{G}$ is fixed within each experiment rather than tuned per Monte Carlo run.

#### 2.3.2. Hidden Variable Prediction and Update

The hidden variable is not modeled as a time-invariant unknown constant. Instead, Equation (22) is used only as a local one-step predictive prior before enforcing the structural relation α=f(x). This prior keeps the previous hidden variable estimate as a temporary prediction, while the uncertainty increment $Q_{k}^{\alpha }$ accounts for the state-dependent variation of $f(x_{k})$ and for higher-order approximation errors

(22) $${\hat{\alpha }}_{k+1|k}={\hat{\alpha }}_{k|k},$$

(23) $$P_{k+1|k}^{\alpha }=P_{k|k}^{\alpha }+Q_{k}^{\alpha },$$

where $Q_{k}^{\alpha }$ is selected as a positive semidefinite design covariance. In the experiments, it is fixed from the local sensitivity of *f* and the expected state uncertainty, i.e.,

(24) $$Q_{k}^{\alpha }=\eta_{\alpha }J_{f,k}Q_{k}J_{f,k}^{T}+q_{\alpha ,\min}I,$$

with small constants $\eta_{\alpha }\geq 0$ and $q_{\alpha ,\min}>0$. This design prevents the hidden variable covariance from collapsing and should be interpreted as local structural uncertainty, not as a claim that $\alpha_{k}$ is an independent random-walk parameter. A possible alternative is to use a structure-informed hidden variable prediction, such as

$${\hat{\alpha }}_{k+1|k}=f\left({\hat{x}}_{k+1|k}\right),$$

or its first-order approximation

$${\hat{\alpha }}_{k+1|k}\approx f\left({\hat{x}}_{k|k}\right)+J_{f,k}\left({\hat{x}}_{k+1|k}-{\hat{x}}_{k|k}\right).$$

Such predictors may better follow nonlinear hidden variable trajectories when the state prediction is accurate. However, they may also transmit state prediction errors directly into the hidden variable prior under strong nonlinearity, high initial uncertainty, or abnormal measurements. Therefore, this study adopts the conservative local prior in Equation (22), followed by measurement update and structural consistency correction. In this design, the prior avoids overcommitting to a possibly inaccurate state prediction, while the final consistency correction still enforces the structural relation α=f(x) after the safety-constrained state update. Investigating adaptive or structure-informed hidden variable prediction models is left as an important future direction. Given ${\hat{x}}_{k+1|k}$, the predicted sensor measurement for the hidden variable update is

(25) $${\hat{z}}_{k+1|k}^{\alpha }=M\left({\hat{x}}_{k+1|k},{\hat{\alpha }}_{k+1|k}\right).$$

The Jacobian matrices with respect to the hidden variable and the state are

(26) $$H_{\alpha }={\left.\frac{\partial M(x,\alpha )}{\partial \alpha }\right|}_{{\hat{x}}_{k+1|k},{\hat{\alpha }}_{k+1|k}},\;H_{x}={\left.\frac{\partial M(x,\alpha )}{\partial x}\right|}_{{\hat{x}}_{k+1|k},{\hat{\alpha }}_{k+1|k}}.$$

To account for state prediction uncertainty during the hidden variable update, the effective measurement noise covariance is written as

(27) $$R_{\mathrm{eff},k}^{\alpha }=R_{k}+H_{x,k}P_{k+1|k}^{x}H_{x,k}^{T}+\Delta_{\alpha x,k},$$

where the subscript *k* emphasizes that the effective covariance is recomputed at every time step. This expression follows from the first-order expansion

(28) $$M\left(x_{k+1},\alpha_{k+1}\right)\approx M\left({\hat{x}}_{k+1|k},{\hat{\alpha }}_{k+1|k}\right)+H_{x,k}\delta x_{k+1}+H_{\alpha ,k}\delta \alpha_{k+1}+\varepsilon_{k+1}.$$

When updating $\alpha_{k+1}$, the state prediction error $H_{x,k}\delta x_{k+1}$ acts as an additional measurement uncertainty. Therefore, its covariance contribution is $H_{x,k}P_{k+1|k}^{x}H_{x,k}^{T}$. The compensation term $\Delta_{\alpha x,k}⪰0$ collects neglected second-order terms and residual state–hidden variable coupling. In the implementation, it is chosen as

(29) $$\Delta_{\alpha x,k}=\eta_{\alpha x}\;\mathrm{diag}\;\left(H_{x,k}P_{k+1|k}^{x}H_{x,k}^{T}\right),\;\eta_{\alpha x}\geq 0,$$

and is kept fixed for a given experimental setting. The innovation covariance and innovation are

(30) $$S_{\alpha ,k}=H_{\alpha ,k}P_{k+1|k}^{\alpha }H_{\alpha ,k}^{T}+R_{\mathrm{eff},k}^{\alpha },$$

(31) $$\nu_{\alpha }=z_{k+1}-{\hat{z}}_{k+1|k}^{\alpha }.$$

Taking the covariance of Equation (31) under the local linearization in Equation (28) gives

(32) $$\mathrm{Cov}\left(\nu_{\alpha }\right)\approx H_{\alpha ,k}P_{k+1|k}^{\alpha }H_{\alpha ,k}^{T}+R_{\mathrm{eff},k}^{\alpha },$$

which yields the innovation covariance in Equation (30). This derivation clarifies that the hidden variable update does not treat the state prediction as deterministic; its uncertainty is included in $R_{\mathrm{eff},k}^{\alpha }$. The raw Kalman gain for the hidden variable update is

(33) $$K_{\alpha }=P_{k+1|k}^{\alpha }H_{\alpha }^{T}S_{\alpha }^{-1}.$$

#### 2.3.3. Safety-Constrained Update

Safe-RTSKF constrains the measurement update using NIS gating, gain clipping, and damping. For the hidden variable update, the NIS statistic is

(34) $${\mathrm{NIS}}_{\alpha }=\nu_{\alpha }^{T}S_{\alpha }^{-1}\nu_{\alpha }.$$

If ${\mathrm{NIS}}_{\alpha }$ exceeds the threshold $\gamma_{\alpha }$, the innovation is scaled by

(35) $$s_{\alpha }=\min\left(1,\sqrt{\frac{\gamma_{\alpha }}{{\mathrm{NIS}}_{\alpha }}}\right).$$

The Kalman gain is clipped using a spectral-norm projection

(36) $$C_{K_{\alpha ,\max}}\left(K_{\alpha }\right)=\left\{\begin{array}{cc} K_{\alpha }, & {∥K_{\alpha }∥}_{2}\leq K_{\alpha ,\max},\\ \frac{K_{\alpha ,\max}}{∥K_{\alpha }{∥}_{2}}K_{\alpha }, & {∥K_{\alpha }∥}_{2}>K_{\alpha ,\max}. \end{array}\right.$$

The effective safe gain is

(37) $$K_{\alpha }^{\mathrm{safe}}=d_{\alpha }s_{\alpha }C_{K_{\alpha ,\max}}\left(K_{\alpha }\right),$$

where $d_{\alpha }\in \left(0,1\right]$ is the damping coefficient. The hidden variable update is then performed as

(38) $${\hat{\alpha }}_{k+1|k+1}^{u}={\hat{\alpha }}_{k+1|k}+K_{\alpha }^{\mathrm{safe}}\nu_{\alpha }.$$

It should be noted that ${\hat{\alpha }}_{k+1|k+1}^{u}$ is only an intermediate measurement-updated proposal. If Equation (38) were used alone with a vanishing $Q_{k}^{\alpha }$, the estimate could behave like a constant-parameter estimator and retain the effect of the initial hidden variable error. Safe-RTSKF avoids this problem by the consistency correction in Equation (44), which repeatedly anchors $\alpha_{k}$ to the state-dependent structural value $f({\hat{x}}_{k})$. Therefore, the final hidden variable estimate is not a pure Kalman estimate of a constant parameter. The covariance is updated using the Joseph form

(39) $$P_{k+1|k+1}^{\alpha ,u}=\left(I-K_{\alpha }^{\mathrm{safe}}H_{\alpha }\right)P_{k+1|k}^{\alpha }{\left(I-K_{\alpha }^{\mathrm{safe}}H_{\alpha }\right)}^{T}+K_{\alpha }^{\mathrm{safe}}R_{\mathrm{eff}}^{\alpha }{\left(K_{\alpha }^{\mathrm{safe}}\right)}^{T}.$$

After the hidden variable update, the state update is performed in a similar manner. The predicted measurement is

(40) $${\hat{z}}_{k+1|k}^{x}=M\left({\hat{x}}_{k+1|k},{\hat{\alpha }}_{k+1|k+1}^{u}\right),$$

and the state direction innovation is

(41) $$\nu_{x}=z_{k+1}-{\hat{z}}_{k+1|k}^{x}.$$

The same NIS gating, spectral-norm gain clipping, damping, and Joseph-form covariance updating are then applied to obtain ${\hat{x}}_{k+1|k+1}^{u}$ and $P_{k+1|k+1}^{x,u}$.

#### 2.3.4. Covariance Projection and Hidden Variable Consistency Correction

To prevent covariance collapse or excessive inflation, Safe-RTSKF applies spectral covariance projection after the update

(42) $$P_{k+1|k+1}^{x}=\Pi_{\left[P_{\min},P_{x,\max}\right]}\left(P_{k+1|k+1}^{x,u}\right),$$

(43) $$P_{k+1|k+1}^{\alpha }=\Pi_{\left[P_{\min},P_{\alpha ,\max}\right]}\left(P_{k+1|k+1}^{\alpha ,u}\right),$$

where $\Pi_{\left[a,b\right]}\left(\cdot \right)$ denotes eigenvalue projection onto the interval [a,b].

Finally, the hidden variable is corrected using the structural relation α=f(x)

(44) $${\hat{\alpha }}_{k+1|k+1}=\rho_{\alpha }{\hat{\alpha }}_{k+1|k+1}^{u}+\left(1-\rho_{\alpha }\right)f\left({\hat{x}}_{k+1|k+1}^{u}\right),$$

where $\rho_{\alpha }\in \left[0,1\right]$ controls the balance between the measurement-updated hidden variable and the structural consistency constraint. A smaller $\rho_{\alpha }$ places more emphasis on the structural relation, whereas a larger $\rho_{\alpha }$ relies more on the recursive hidden variable update.

The corresponding hidden variable covariance is approximated as

(45) $$\begin{array}{cc} P_{k+1|k+1}^{\alpha }\approx & \rho_{\alpha }^{2}P_{k+1|k+1}^{\alpha ,u}+{\left(1-\rho_{\alpha }\right)}^{2}J_{f}P_{k+1|k+1}^{x,u}J_{f}^{T}\\ \; & +\rho_{\alpha }\left(1-\rho_{\alpha }\right)\left(P_{k+1|k+1}^{\alpha x,u}J_{f}^{T}+J_{f}{\left(P_{k+1|k+1}^{\alpha x,u}\right)}^{T}\right), \end{array}$$

where

(46) $$J_{f}={\left.\frac{\partial f(x)}{\partial x}\right|}_{{\hat{x}}_{k+1|k+1}^{u}},$$

and $P_{k+1|k+1}^{\alpha x,u}$ denotes the cross-covariance between the measurement-updated hidden variable and the updated state. When this cross-covariance is not explicitly propagated, a conservative implementation can set $P_{k+1|k+1}^{\alpha x,u}=0$ and increase the diagonal inflation before projection. The revised expression makes the approximation explicit and avoids implicitly assuming that the two terms are always uncorrelated.

This consistency correction is the key difference between Safe-RTSKF and a conventional robustified Kalman update. It explicitly suppresses the drift of the hidden variable away from its state-dependent structural definition.

#### 2.3.5. Complete Safe-RTSKF Pseudocode

For clarity, Table 2 summarizes the complete execution order of the proposed method. The hidden variable update is performed first, followed by the state update, covariance projection, and structural consistency correction.

#### 2.3.6. Mathematical Rationale Compared with a Direct Kalman Update

The proposed method is not intended to replace the Kalman filter in linear Gaussian systems. Its advantage appears when the system dynamics or measurements contain products or structurally coupled nonlinear functions. For two random variables *a* and *b*, the optimal mean square estimate of their product under information set Y is

(47) E[ab|Y]=E[a|Y]E[b|Y]+Cov(a,b|Y).

Thus, the product of two separate estimates ignores the conditional covariance term and is generally not equal to the minimum mean square estimate of the product. In the one-dimensional model $x_{k+1}=x_{k}\alpha_{k}+w_{k}$, a direct Kalman-type update based only on ${\hat{x}}_{k|k}$ and ${\hat{\alpha }}_{k|k}$ approximates the nonlinear product by ${\hat{x}}_{k|k}{\hat{\alpha }}_{k|k}$, while Safe-RTSKF additionally propagates the product variance in Equation (20) and constrains the hidden variable estimate through $f(\hat{x})$.

The additional computational cost is also acknowledged. If *n* is the state dimension, *r* is the hidden variable dimension, and *m* is the measurement dimension, the dominant matrix inversions and covariance updates of Safe-RTSKF have complexity

(48) $$O(n^{3}+r^{3}+m^{3}),$$

whereas a standard Kalman-type filter without hidden variables is typically dominated by $O(n^{3}+m^{3})$. Therefore, Safe-RTSKF is most useful when the hidden variable dimension is moderate and the stability gain compensates for the additional cost. The runtime experiments in Section 3.7 are included for this reason.

### 2.4. Theoretical Analysis

This subsection provides a local theoretical explanation of why the proposed safety-constrained update and hidden variable consistency correction can improve recursive stability. The analysis is not intended to establish global optimality or global convergence. Instead, it focuses on three key aspects: the error bound of the hidden variable consistency correction, the boundedness of the safety-constrained update, and the boundedness of covariance projection.

**Assumption 1.** 
*The true state and hidden variable satisfy the structural relation*

(49) $$\alpha^{*}=f\left(x^{*}\right).$$

*The function f(·) is Lipschitz continuous in the considered local region; that is, there exists a constant $L_{f}>0$ such that*

(50) $$∥f\left(x_{1}\right)-f\left(x_{2}\right)∥\leq L_{f}∥x_{1}-x_{2}∥,\;\forall x_{1},x_{2}.$$

Let the hidden variable error before consistency correction and the state estimation error after update be defined as

(51) $$e_{\alpha }^{u}={\hat{\alpha }}^{u}-\alpha^{*},$$

(52) $$e_{x}={\hat{x}}^{u}-x^{*}.$$

The consistency-corrected hidden variable is

(53) $${\hat{\alpha }}^{c}=\rho_{\alpha }{\hat{\alpha }}^{u}+\left(1-\rho_{\alpha }\right)f\left({\hat{x}}^{u}\right),\;0\leq \rho_{\alpha }\leq 1.$$

The corresponding corrected hidden variable error is

(54) $$e_{\alpha }^{c}={\hat{\alpha }}^{c}-\alpha^{*}.$$

**Theorem 1.** 
***Hidden variable consistency error bound.*** *Under the above assumption, the consistency-corrected hidden variable error satisfies*

(55) $$∥e_{\alpha }^{c}∥\leq \rho_{\alpha }∥e_{\alpha }^{u}∥+\left(1-\rho_{\alpha }\right)L_{f}∥e_{x}∥.$$

**Proof.**  From Equations (49) and (53), we have

(56) $$\begin{array}{cc} e_{\alpha }^{c}\; & ={\hat{\alpha }}^{c}-\alpha^{*}\\ \; & =\rho_{\alpha }{\hat{\alpha }}^{u}+\left(1-\rho_{\alpha }\right)f\left({\hat{x}}^{u}\right)-f\left(x^{*}\right)\\ \; & =\rho_{\alpha }\left({\hat{\alpha }}^{u}-f\left(x^{*}\right)\right)+\left(1-\rho_{\alpha }\right)\left(f\left({\hat{x}}^{u}\right)-f\left(x^{*}\right)\right)\\ \; & =\rho_{\alpha }e_{\alpha }^{u}+\left(1-\rho_{\alpha }\right)\left(f\left({\hat{x}}^{u}\right)-f\left(x^{*}\right)\right). \end{array}$$

Taking the norm on both sides and using the triangle inequality gives

(57) $$∥e_{\alpha }^{c}∥\leq \rho_{\alpha }∥e_{\alpha }^{u}∥+\left(1-\rho_{\alpha }\right)∥f\left({\hat{x}}^{u}\right)-f\left(x^{*}\right)∥.$$

By the Lipschitz condition in Equation (50),

(58) $$∥f\left({\hat{x}}^{u}\right)-f\left(x^{*}\right)∥\leq L_{f}∥{\hat{x}}^{u}-x^{*}∥=L_{f}∥e_{x}∥.$$

Substituting Equation (58) into Equation (57) yields Equation (55). □

**Corollary 1.** 
***Local contraction condition.*** *If there exists a constant η∈[0,1) such that*

(59) $$L_{f}∥e_{x}∥\leq \eta ∥e_{\alpha }^{u}∥,$$

*then*

(60) $$∥e_{\alpha }^{c}∥\leq \left[\rho_{\alpha }+\left(1-\rho_{\alpha }\right)\eta \right]∥e_{\alpha }^{u}∥<∥e_{\alpha }^{u}∥.$$

*Thus, when the state estimation error is locally controlled, the consistency correction can reduce the hidden variable error.*

**Proposition 1.** 
***Boundedness of safety-constrained update.*** *Consider a state update of the form*

(61) $$\Delta x_{k}=K_{x}^{\mathrm{safe}}\nu_{x},$$

*where*

(62) $$K_{x}^{\mathrm{safe}}=d_{x}s_{x}C_{K_{x,\max}}\left(K_{x}\right),\;0<d_{x}\leq 1,\;0<s_{x}\leq 1.$$

*Assume that the innovation covariance $S_{x}$ is positive-definite and that the NIS-gated innovation satisfies*

(63) $$\nu_{x}^{T}S_{x}^{-1}\nu_{x}\leq \gamma_{x}.$$

*Then the single-step update is bounded by*

(64) $${∥\Delta x_{k}∥}_{2}\leq d_{x}K_{x,\max}\sqrt{\gamma_{x}\lambda_{\max}\left(S_{x}\right)}.$$

**Proof.**  Since $S_{x}$ is positive-definite,

(65) $${∥\nu_{x}∥}_{2}^{2}\leq \lambda_{\max}\left(S_{x}\right)\nu_{x}^{T}S_{x}^{-1}\nu_{x}.$$

Using Equation (63), we obtain

(66) $${∥\nu_{x}∥}_{2}\leq \sqrt{\gamma_{x}\lambda_{\max}\left(S_{x}\right)}.$$

The spectral-norm gain projection satisfies

(67) $${∥C_{K_{x,\max}}\left(K_{x}\right)∥}_{2}\leq K_{x,\max}.$$

Therefore,

(68) $${∥K_{x}^{\mathrm{safe}}∥}_{2}\leq d_{x}K_{x,\max}.$$

Combining Equations (66) and (68) yields

(69) $${∥\Delta x_{k}∥}_{2}\leq {∥K_{x}^{\mathrm{safe}}∥}_{2}{∥\nu_{x}∥}_{2}\leq d_{x}K_{x,\max}\sqrt{\gamma_{x}\lambda_{\max}\left(S_{x}\right)}.$$

□

**Proposition 2.** 
***Boundedness of covariance projection.*** *Let $\Pi_{\left[P_{\min},P_{\max}\right]}\left(\cdot \right)$ denote the spectral projection that clips all eigenvalues of a symmetric covariance matrix to the interval $\left[P_{\min},P_{\max}\right]$, where $0<P_{\min}<P_{\max}$. Then the projected covariance matrix satisfies*

(70) $$P_{\min}I⪯\Pi_{\left[P_{\min},P_{\max}\right]}\left(P\right)⪯P_{\max}I.$$

*Consequently, the projected state and hidden variable covariance matrices remain bounded*

(71) $$P_{\min}I⪯P_{k|k}^{x}⪯P_{x,\max}I,\;P_{\min}I⪯P_{k|k}^{\alpha }⪯P_{\alpha ,\max}I.$$

**Proof.**  For any symmetric covariance matrix P, let its eigenvalue decomposition be

(72) $$P=U\Lambda U^{T}.$$

The spectral projection replaces each eigenvalue $\lambda_{i}$ by

(73) $${\tilde{\lambda }}_{i}=\min\left\{\max\left(\lambda_{i},P_{\min}\right),P_{\max}\right\}.$$

Thus, $P_{\min}\leq {\tilde{\lambda }}_{i}\leq P_{\max}$ for all *i*. The projected matrix therefore satisfies Equation (70). Applying this result to the state and hidden variable covariance matrices gives Equation (71). □

**Remark 1.** 
*The above results are local boundedness and consistency results rather than global convergence guarantees. The consistency correction can reduce hidden variable drift when the state estimation error is locally controlled, while NIS gating, spectral-norm gain clipping, and covariance projection limit unsafe innovation-driven updates and covariance inflation. These properties explain why Safe-RTSKF can improve the recursive stability of Raw RTSKF in strongly nonlinear sensor estimation problems.*

#### Connection Between Local Theory and Empirical Stability

The theoretical results above should be interpreted as local stability explanations rather than global convergence guarantees. In practical recursive filtering, the local contraction condition in Corollary 1 is expected to hold when three conditions are approximately satisfied. First, the state estimate after the safety-constrained update remains in a local neighborhood of the true state, so that the Lipschitz approximation of the structural mapping f(·) is meaningful. Second, the measurement innovation is prevented from producing an excessively large update, which is enforced by NIS gating, spectral-norm gain clipping, and update damping. Third, the covariance matrices remain numerically bounded through spectral projection, preventing the filter from becoming overconfident or excessively uncertain.

Under these conditions, the consistency correction reduces the hidden variable error whenever the state-induced structural error $L_{f}∥e_{x}∥$ is smaller than a fixed fraction of the pre-correction hidden variable error $∥e_{\alpha }^{u}∥$. This provides a local mechanism for reducing hidden variable drift. The Monte Carlo RMSE reductions should therefore not be interpreted as a direct quantitative consequence of the bound alone. Rather, the theoretical bound explains why repeated local consistency corrections can suppress hidden variable error accumulation, while the empirical results demonstrate that this local mechanism remains effective over many recursive filtering steps.

### 2.5. Safe-EKF Extension for Range–Bearing Sensor Tracking

Although Safe-RTSKF is designed for strongly nonlinear systems with multiplicative hidden variable structures, the safety update mechanism can also be transferred to conventional nonlinear Kalman filtering. Range–bearing target tracking is a representative nonlinear sensor estimation problem and has been widely studied using EKF, UKF, CKF, and their robust or adaptive variants [24,25,27,35]. To evaluate this transferability in a sensor tracking scenario, this study constructs a Safe-EKF for range–bearing target tracking under outlier-contaminated measurements. This extension does not use the hidden variable decomposition of RTSKF; instead, it applies NIS gating, spectral-norm gain clipping, Joseph-form covariance updating, and covariance projection to the standard EKF update.

The target state is defined as

(74) $$x_{k}={\left[p_{x,k},p_{y,k},v_{x,k},v_{y,k}\right]}^{T},$$

where $\left(p_{x,k},p_{y,k}\right)$ denotes the two-dimensional position and $\left(v_{x,k},v_{y,k}\right)$ denotes the velocity. The constant velocity motion model is

(75) $$x_{k+1}=Fx_{k}+w_{k},$$

where

(76) $$F=\left[\begin{array}{cccc} 1 & 0 & \Delta t & 0\\ 0 & 1 & 0 & \Delta t\\ 0 & 0 & 1 & 0\\ 0 & 0 & 0 & 1 \end{array}\right].$$

The process noise covariance is modeled as

(77) $$Q=G_{a}\left(\sigma_{a}^{2}I_{2}\right)G_{a}^{T},$$

with

(78) $$G_{a}=\left[\begin{array}{cc} \frac{1}{2}\Delta t^{2} & 0\\ 0 & \frac{1}{2}\Delta t^{2}\\ \Delta t & 0\\ 0 & \Delta t \end{array}\right],$$

where $\sigma_{a}$ denotes the acceleration noise standard deviation.

The range–bearing sensor measurement is

(79) $$z_{k}=h\left(x_{k}\right)+\varepsilon_{k},$$

where

(80) $$h\left(x_{k}\right)=\left[\begin{array}{c} r_{k}\\ \theta_{k} \end{array}\right]=\left[\begin{array}{c} \sqrt{p_{x,k}^{2}+p_{y,k}^{2}}\\ \mathrm{atan}2(p_{y,k},p_{x,k}) \end{array}\right].$$

The measurement noise covariance is

(81) $$R=\mathrm{diag}(\sigma_{r}^{2},\sigma_{\theta }^{2}),$$

where $\sigma_{r}$ and $\sigma_{\theta }$ are the range and bearing noise standard deviations, respectively. In the outlier-contaminated scenario, additional abnormal perturbations are injected into a small proportion of measurements to evaluate robustness against unsafe innovations.

For the EKF update, the measurement Jacobian is

(82) $$H_{k}={\left.\frac{\partial h(x)}{\partial x}\right|}_{{\hat{x}}_{k|k-1}}=\left[\begin{array}{cccc} \frac{{\hat{p}}_{x}}{\hat{r}} & \frac{{\hat{p}}_{y}}{\hat{r}} & 0 & 0\\ -\frac{{\hat{p}}_{y}}{{\hat{r}}^{2}} & \frac{{\hat{p}}_{x}}{{\hat{r}}^{2}} & 0 & 0 \end{array}\right],$$

where $\hat{r}=\sqrt{{\hat{p}}_{x}^{2}+{\hat{p}}_{y}^{2}}$. The predicted measurement and innovation are

(83) $${\hat{z}}_{k|k-1}=h\left({\hat{x}}_{k|k-1}\right),$$

(84) $$\nu_{k}=z_{k}-{\hat{z}}_{k|k-1}.$$

Because the second component of the measurement is an angle, the bearing innovation is wrapped into [−π,π]

(85) $$\nu_{\theta ,k}\leftarrow {\mathrm{wrap}}_{\pi }\left(\nu_{\theta ,k}\right).$$

The innovation covariance and raw Kalman gain are

(86) $$S_{k}=H_{k}P_{k|k-1}H_{k}^{T}+R,$$

(87) $$K_{k}=P_{k|k-1}H_{k}^{T}S_{k}^{-1}.$$

Safe-EKF first computes the NIS statistic

(88) $${\mathrm{NIS}}_{k}=\nu_{k}^{T}S_{k}^{-1}\nu_{k}.$$

If the innovation is abnormal, the update is scaled by

(89) $$s_{k}=\min\left(1,\sqrt{\frac{\gamma }{{\mathrm{NIS}}_{k}}}\right),$$

where γ is the NIS gating threshold. The Kalman gain is then projected by the spectral-norm clipping operator

(90) $$K_{k}^{\mathrm{clip}}=C_{K_{\max}}\left(K_{k}\right),$$

where $C_{K_{\max}}\left(\cdot \right)$ is defined in the same way as Equation (36). The final safe gain is

(91) $$K_{k}^{\mathrm{safe}}=ds_{k}K_{k}^{\mathrm{clip}},$$

where d∈(0,1] is the damping coefficient.

The Safe-EKF state update is

(92) $${\hat{x}}_{k|k}={\hat{x}}_{k|k-1}+K_{k}^{\mathrm{safe}}\nu_{k}.$$

The covariance is updated using the Joseph form

(93) $$P_{k|k}=\left(I-K_{k}^{\mathrm{safe}}H_{k}\right)P_{k|k-1}{\left(I-K_{k}^{\mathrm{safe}}H_{k}\right)}^{T}+K_{k}^{\mathrm{safe}}R{\left(K_{k}^{\mathrm{safe}}\right)}^{T}.$$

Finally, covariance spectral projection is applied

(94) $$P_{k|k}\leftarrow \Pi_{\left[P_{\min},P_{\max}\right]}\left(P_{k|k}\right).$$

This Safe-EKF extension is used only to examine whether the proposed safety update idea can improve the robustness of a conventional nonlinear Kalman filter in sensor tracking problems. It should not be interpreted as a direct application of the hidden variable RTSKF structure. In this paper, Safe-RTSKF is evaluated on strongly nonlinear hidden variable systems, whereas Safe-EKF is used as an application-oriented extension for range–bearing and coordinated turn tracking under outlier-contaminated measurements.

## 3. Results

### 3.1. Experimental Settings and Baseline Methods

This section evaluates the proposed Safe-RTSKF and the safety update extension from three perspectives: strongly nonlinear hidden variable systems, range–bearing sensor tracking, and coordinated turn tracking. The first group of experiments is designed to examine whether Safe-RTSKF can improve the recursive stability of Raw RTSKF in multiplicative hidden variable systems. The second and third groups of experiments evaluate whether the safety update mechanism can improve the robustness of EKF-type sensor tracking under outlier-contaminated measurements and maneuvering dynamics.

All Monte Carlo experiments were conducted using the same random seed protocol for all compared methods. For the *i*-th Monte Carlo run, the random seed was set as

(95) $${\mathrm{seed}}_{i}=2025+i,\;i=0,1,\ldots ,N_{\mathrm{MC}}-1.$$

For each Monte Carlo index, all methods used the same true trajectory, process noise sequence, and sensor measurement sequence. This setting ensures that the comparison reflects the difference among filtering algorithms rather than random variations in data generation.

For the strongly nonlinear hidden variable experiments, the trajectory length was set to

(96) T=100.

Unless otherwise specified, the extended baseline comparison, ablation study, and parameter sensitivity analysis used $N_{\mathrm{MC}}=300$ Monte Carlo runs. The range–bearing target tracking and coordinated turn benchmark used $N_{\mathrm{MC}}=100$ Monte Carlo runs.

The compared methods include EKF, UKF, CKF, Robust UKF, Adaptive UKF, $H_{\infty }$-EKF, Bootstrap PF, Raw RTSKF, Safe-RTSKF, and Safe-EKF. These baselines cover classical Kalman filtering, deterministic sampling-based nonlinear filtering, robust/adaptive filtering, and sampling-based Bayesian filtering [1,5,6,7,8,9,11,13]. The baseline settings are summarized in Table 3. EKF, UKF, and CKF are used as standard nonlinear Kalman filtering baselines. Robust UKF, Adaptive UKF, and $H_{\infty }$-EKF are included as robust or adaptive filtering baselines. Bootstrap PF is included as a sampling-based nonlinear filtering baseline. Raw RTSKF is used to directly evaluate the stability improvement brought by Safe-RTSKF. Bootstrap PF was selected as the particle filter baseline because it is the most standard and transparent sampling-based Bayesian filtering method. It uses prior proposal propagation, likelihood-based weight updating, and systematic resampling, and therefore provides a clear reference for comparing recursive Kalman-type filters with a basic particle approximation. More advanced PF variants, such as robust PF, auxiliary PF, unscented PF, and Rao–Blackwellized PF, may achieve better performance under outliers or special model structures, but they introduce additional design choices such as robust likelihood functions, auxiliary proposal distributions, conditional linear–Gaussian decompositions, or problem-specific marginalization. Since the purpose of this study is to evaluate the proposed safety-constrained Kalman-type framework rather than to optimize particle filtering, Bootstrap PF is used as a transparent baseline, while advanced PF variants are left for future comparative studies.

To improve reproducibility, the main simulation settings of the one-, two-, and four-dimensional strongly nonlinear systems are summarized in Table 4. All parameter values were fixed before the Monte Carlo runs and were not selected separately for individual random seeds. For the same Monte Carlo index, all compared methods used the same true trajectory, process noise sequence, and measurement noise sequence.

The main evaluation metrics are root mean square error (RMSE), mean absolute error (MAE), average covariance trace, divergence rate, and runtime. For a state estimate ${\hat{x}}_{k}^{\left(i\right)}$ and the corresponding true state $x_{k}^{\left(i\right)}$ in the *i*-th Monte Carlo run, the RMSE is computed as

(97) $${\mathrm{RMSE}}^{\left(i\right)}=\sqrt{\frac{1}{T}\sum_{k=1}^{T}{∥{\hat{x}}_{k}^{\left(i\right)}-x_{k}^{\left(i\right)}∥}_{2}^{2}}.$$

The MAE is computed as

(98) $${\mathrm{MAE}}^{\left(i\right)}=\frac{1}{T}\sum_{k=1}^{T}{∥{\hat{x}}_{k}^{\left(i\right)}-x_{k}^{\left(i\right)}∥}_{1}.$$

For target tracking experiments, the position RMSE is computed using only the two-dimensional position components

(99) $${\mathrm{RMSE}}_{p}^{\left(i\right)}=\sqrt{\frac{1}{T}\sum_{k=1}^{T}\left[{\left({\hat{p}}_{x,k}^{\left(i\right)}-p_{x,k}^{\left(i\right)}\right)}^{2}+{\left({\hat{p}}_{y,k}^{\left(i\right)}-p_{y,k}^{\left(i\right)}\right)}^{2}\right]}.$$

A Monte Carlo run was regarded as divergent if any of the following conditions occurred: the state estimate contained NaN or Inf values; the absolute value of the one-dimensional state estimate exceeded $10^{5}$; the Euclidean norm of a multi-dimensional state estimate exceeded $10^{5}$; or the covariance matrix became severely ill-conditioned and could not be recovered by positive-definite correction. The threshold $10^{5}$ was chosen as a numerical blow-up threshold rather than a physical range limit. In all simulated systems, the nominal state magnitude remains several orders of magnitude smaller than $10^{5}$. Therefore, once an estimate exceeds this value, the corresponding trajectory no longer represents a meaningful state estimate but a numerical divergence event. We also verified that using thresholds in the range $10^{4}$–$10^{6}$ does not change the qualitative ranking of the methods, because divergent Raw RTSKF trajectories usually grow far beyond these values. The divergence rate was computed as

(100) $$\mathrm{Divergence}\;\mathrm{Rate}=\frac{N_{\mathrm{div}}}{N_{\mathrm{MC}}}\times 100\%,$$

where $N_{\mathrm{div}}$ is the number of divergent Monte Carlo runs.

In addition to mean and standard deviation, robust statistics including the median, interquartile range (IQR), and bootstrap 95% confidence interval were also reported for key experiments. Paired statistical tests were conducted using the per-run RMSE records. For two compared methods, the paired difference was defined as

(101) $$d_{i}={\mathrm{RMSE}}_{\mathrm{proposed},i}-{\mathrm{RMSE}}_{\mathrm{baseline},i}.$$

If $d_{i}<0$, the proposed method achieved a lower error in the *i*-th Monte Carlo run. Normality was first examined using the Shapiro–Wilk test. If the paired differences were approximately normal, a paired *t*-test was used; otherwise, the Wilcoxon signed-rank test was applied. Holm–Bonferroni correction was used for multiple comparisons.

Runtime was measured using the same implementation environment for all methods. The average runtime per Monte Carlo run was reported to evaluate the accuracy–runtime trade-off. This is particularly important for sensor state estimation because real-time deployment usually requires both estimation robustness and computational efficiency.

### 3.2. Results on Strongly Nonlinear Hidden Variable Systems

This subsection reports the results on one-dimensional, two-dimensional, and four-dimensional strongly nonlinear systems with hidden variable structures. These experiments are designed to evaluate whether Safe-RTSKF can improve the recursive stability of Raw RTSKF under strong nonlinearity and high uncertainty. The purpose is not to show that Safe-RTSKF is universally superior to all nonlinear filters, but to examine whether the proposed safety constraints and hidden variable consistency correction can reduce the instability of Raw RTSKF while maintaining a competitive accuracy–runtime trade-off.

Table 5 shows the comparison results on the one-dimensional strongly nonlinear system. Bootstrap PF achieved the lowest RMSE, indicating that particle approximation can be highly effective in this low-dimensional, strongly nonlinear scenario. Safe-RTSKF achieved an RMSE of 0.0761 with zero divergence, which was lower than those of EKF, CKF, $H_{\infty }$-EKF, and Raw RTSKF. In contrast, Raw RTSKF suffered from severe instability, with an RMSE of 10,168.3269 and a divergence rate of 89.00%. These results indicate that Safe-RTSKF substantially improves the stability of Raw RTSKF and provides competitive performance among non-particle recursive filters.

**Figure 1:**
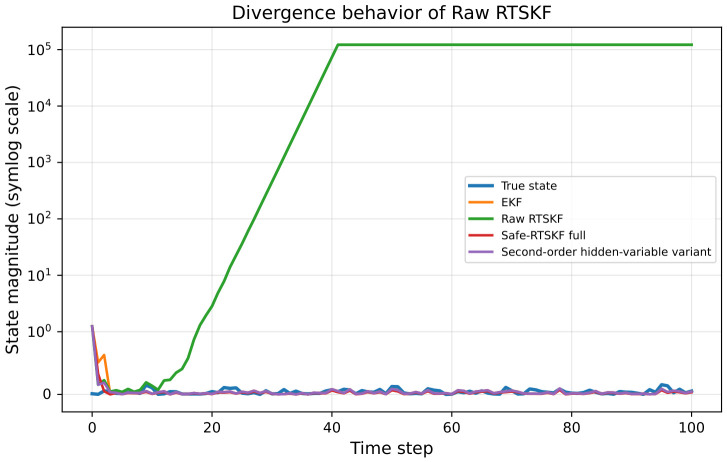
Divergence behavior of Raw RTSKF in the one-dimensional strongly nonlinear hidden variable system. The vertical axis uses a symmetric logarithmic scale to highlight recursive error amplification. Raw RTSKF rapidly grows to the numerical divergence region, whereas EKF, Safe-RTSKF full, and the second-order hidden variable variant remain bounded.

Figure 1 further illustrates the divergence behavior of Raw RTSKF in the one-dimensional strongly nonlinear system.

**Figure 2:**
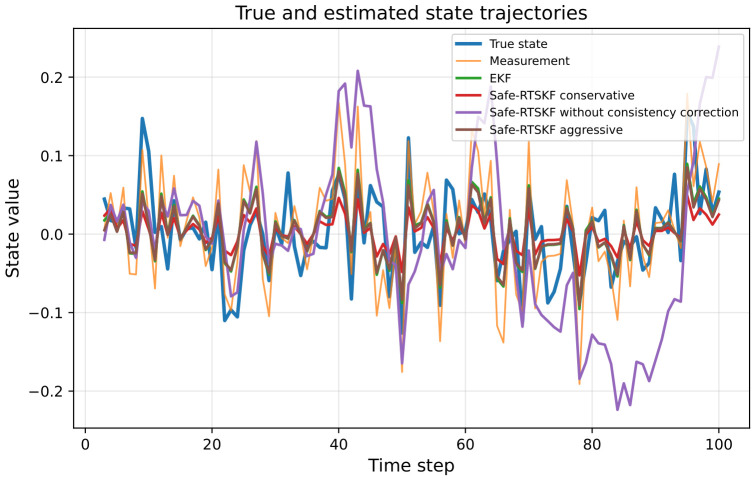
True and estimated state trajectories in the one-dimensional strongly nonlinear hidden variable system. The first three transient steps are omitted to better visualize the steady recursive tracking behavior. The variant without consistency correction shows visible drift in later time steps, while the consistency-corrected Safe-RTSKF variants remain closer to the true state.

The state trajectory comparison in Figure 2 shows that the consistency-corrected Safe-RTSKF variants remain closer to the true state than the variant without consistency correction.

Table 6 reports the results on the two-dimensional coupled strongly nonlinear system. In this case, EKF achieved the lowest RMSE, and UKF, Robust UKF, and Bootstrap PF also performed well. Safe-RTSKF achieved an RMSE of 0.0589 with zero divergence, which was close to the best-performing methods and lower than those of Adaptive UKF, CKF, $H_{\infty }$-EKF, and Raw RTSKF. Compared with Raw RTSKF, Safe-RTSKF reduced the RMSE from 0.0900 to 0.0589. This result shows that Safe-RTSKF does not always achieve the lowest RMSE, but it consistently improves the Raw RTSKF structure and reaches a performance level close to mainstream nonlinear Kalman filters.

#### Additional Comparison with Augmented-State Baselines

To address whether the two-stage structure provides advantages beyond a direct augmented-state formulation, additional Augmented EKF and Augmented UKF baselines were implemented. These filters jointly estimate the augmented vector

$${\bar{x}}_{k}={\left[x_{k}^{T},\alpha_{k}^{T}\right]}^{T}$$

under a separate augmented-state validation setting. Within this additional comparison, all methods use the same Monte Carlo seeds, true trajectories, process noise sequences, and measurement noise sequences. Therefore, the comparison in Table 7 is internally fair among Augmented EKF, Augmented UKF, Raw RTSKF, and Safe-RTSKF. However, the numerical RMSE values in Table 7 are not intended to be directly identical to those in Table 5 and Table 6, because Table 5 and Table 6 report the main extended-baseline experiments, whereas Table 7 reports an additional augmented-state validation experiment with its own implementation setting. The results are summarized in Table 7. In the one-dimensional setting, all methods remained stable, and Raw RTSKF achieved the lowest RMSE. In the two-dimensional coupled setting, Safe-RTSKF achieved the lowest RMSE among the compared augmented-state and two-stage filters, with substantially lower runtime than Augmented EKF and Augmented UKF. These results indicate that augmented-state filtering is a meaningful alternative, but it does not consistently outperform the proposed safety-constrained two-stage formulation. The advantage of Safe-RTSKF is more evident in terms of computational efficiency and in settings where Raw RTSKF is prone to hidden variable drift or numerical instability.

This additional comparison is used only to examine the augmented-state modeling alternative and does not replace the main extended-baseline comparison in Table 5 and Table 6. Therefore, the ranking and relative behavior within Table 7 should be interpreted independently of the absolute RMSE values reported in the main comparison tables.

To further examine the scalability of Safe-RTSKF, a four-dimensional cyclically coupled system was constructed. The state variables are coupled through a cyclic hidden variable structure, and the measurement model is also nonlinear. The results are shown in Table 8. EKF achieved the lowest RMSE, followed by UKF and CKF. Safe-RTSKF achieved an RMSE of 0.0860, which was slightly higher than EKF, UKF, and CKF, but lower than Bootstrap PF and Raw RTSKF. Compared with Raw RTSKF, Safe-RTSKF reduced the RMSE from 0.1219 to 0.0860, corresponding to a reduction of approximately 29.43%. This confirms that the proposed safety constraints and consistency correction remain effective in a higher-dimensional hidden variable system.

The four-dimensional results also highlight the accuracy–runtime trade-off. Bootstrap PF required 9.2302 s per Monte Carlo run, whereas Safe-RTSKF required only 0.0552 s. Thus, Bootstrap PF was approximately 167 times slower than Safe-RTSKF in this experiment. Although PF can be advantageous in low-dimensional strongly nonlinear systems, its computational cost becomes much higher as the state dimension and particle number increase. Safe-RTSKF therefore provides a practical recursive alternative when both stability and runtime efficiency are important.

Overall, these strongly nonlinear system experiments support three observations. First, Safe-RTSKF substantially improves Raw RTSKF across all tested hidden variable systems. Second, Safe-RTSKF is not always the lowest-RMSE method; EKF, UKF, CKF, or PF may be better in some specific settings. Third, Safe-RTSKF provides a useful stability enhancement mechanism for Raw RTSKF while maintaining a low-to-moderate computational cost.

### 3.3. Ablation Study

To examine the individual contribution of each safety module, an ablation study was conducted on the one-dimensional strongly nonlinear hidden variable system. Six versions were compared: Raw RTSKF, Raw RTSKF with NIS gating, Raw RTSKF with NIS gating and gain clipping, Raw RTSKF with additional update damping, Raw RTSKF with covariance bounding, and the full Safe-RTSKF with hidden variable consistency correction. The results are summarized in Table 9.

**Figure 3:**
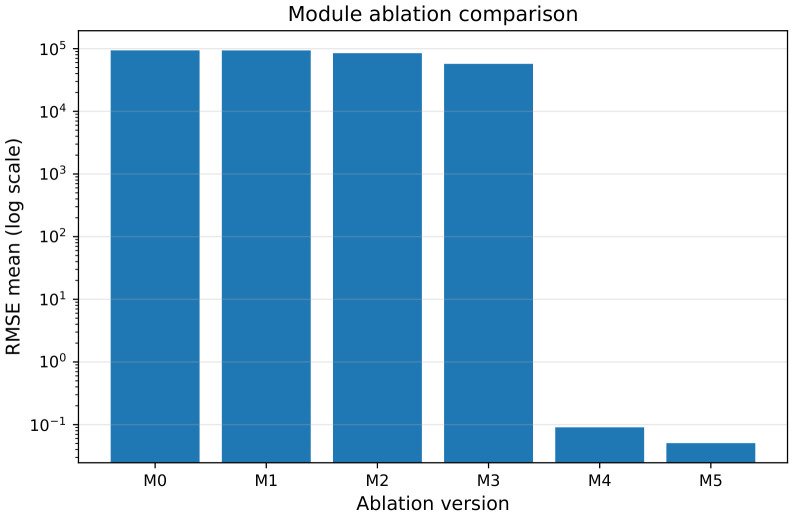
Ablation comparison of the proposed safety modules. M0 denotes Raw RTSKF; M1 adds NIS gating; M2 adds gain clipping; M3 adds update damping; M4 adds covariance bounding; and M5 denotes the full Safe-RTSKF with hidden variable consistency correction. The logarithmic RMSE scale shows that covariance bounding removes numerical divergence, while the full Safe-RTSKF further reduces the estimation error.

Figure 3 visually confirms that covariance projection and hidden variable consistency correction are the two most important components for stabilizing Raw RTSKF.

The ablation results show that Raw RTSKF is highly unstable in the strongly nonlinear setting. The RMSE mean of M0 reached 10,327.1295, and the divergence rate was 93.00%. Adding only NIS gating did not significantly improve the result, indicating that innovation gating alone is insufficient to eliminate the hidden variable drift and covariance inflation of Raw RTSKF. After gain clipping was added, the average covariance trace decreased, but the RMSE and divergence rate remained high. This suggests that limiting the Kalman gain can suppress some extreme correction steps but cannot fully stabilize the recursive two-stage structure.

The damping module further reduced the RMSE from 10,357.6010 to 6483.4219 and decreased the divergence rate from 93.00% to 88.33%. However, the filter still diverged in most Monte Carlo runs. The most significant improvement occurred after covariance bounding was introduced. The RMSE mean decreased sharply to 0.1853, and the divergence rate dropped to 0.00%. This result indicates that covariance spectral projection is the key module for preventing numerical divergence in this difficult hidden variable system.

The full Safe-RTSKF further reduced the RMSE mean from 0.1853 to 0.0758 and reduced the RMSE standard deviation from 0.3125 to 0.0116. This improvement is attributed to the hidden variable consistency correction, which pulls the updated hidden variable back toward its structural relation with the state. Therefore, the ablation study indicates that covariance bounding mainly prevents divergence, whereas hidden variable consistency correction mainly improves estimation accuracy and Monte Carlo stability after numerical stability has been achieved.

### 3.4. Parameter Sensitivity Analysis

A one-factor parameter sensitivity analysis was conducted to evaluate whether the performance of Safe-RTSKF depends on a single accidental parameter combination. In each experiment, only one safety parameter was varied while all other parameters were fixed. The tested parameters include the damping coefficients $d_{x}$ and $d_{\alpha }$, the hidden variable consistency weight $\rho_{\alpha }$, the NIS gating thresholds $\gamma_{x}$ and $\gamma_{\alpha }$, the hidden variable covariance upper bound $P_{\alpha ,\max}$, and the state covariance upper bound $P_{x,\max}$. The sensitivity analysis was performed on both one-dimensional and two-dimensional strongly nonlinear systems.

The summarized single-factor results are listed in Table 10. To avoid ambiguity, the complete candidate values used in each one-factor sweep are also reported in the table.

To further quantify parameter sensitivity, the coefficient of variation (CV) of RMSE was computed across different values of each parameter. The results are reported in Table 11. In the one-dimensional system, the damping coefficient had the largest RMSE variability, with a CV of 0.1707. This confirms that the damping coefficient is the most sensitive safety parameter in the one-dimensional system. The NIS gating threshold, covariance bounds, and consistency weight showed much smaller RMSE variations and were therefore classified as highly stable.

In the two-dimensional system, all tested parameters showed moderate RMSE variability. This indicates that the two-dimensional coupled system is somewhat more sensitive to safety parameter changes than the one-dimensional system. Nevertheless, the RMSE variation remained controlled, and no divergence was observed in the tested parameter ranges. These results suggest that the stability of Safe-RTSKF is not produced by a single carefully tuned parameter combination. Instead, the safety constraints and hidden variable consistency correction provide a relatively robust stability enhancement mechanism over a range of parameter settings.

Overall, the sensitivity analysis shows that the damping coefficient and the consistency weight are the most influential parameters for estimation accuracy, whereas the NIS gating thresholds and covariance bounds primarily contribute to update safety and numerical stability. In practical applications, the damping coefficient can be selected according to the expected severity of abnormal innovations, while $\rho_{\alpha }$ can be adjusted according to the reliability of the structural relation α=f(x).

#### Practical Parameter Selection Guideline

The tested parameter ranges were selected around the fixed values used in the main experiments to examine whether the proposed method depends on a narrow accidental setting. In practical applications, the NIS gate threshold γ can be initialized from a chi-square quantile with degrees of freedom equal to the measurement dimension and then slightly enlarged when the assumed measurement noise covariance is uncertain. The gain clipping bound $K_{\max}$ should be chosen large enough not to affect normal updates, but small enough to suppress abnormal high-gain corrections under outliers. The damping coefficient *d* controls the responsiveness–robustness trade-off: smaller values are preferable under frequent abnormal measurements, whereas values close to one are suitable for clean measurements. The consistency coefficient $\rho_{\alpha }$ should be smaller when the structural relation α=f(x) is reliable, and larger when the structural model may be biased. The covariance bounds $P_{\min}$, $P_{\max}^{x}$, and $P_{\max}^{\alpha }$ should be selected according to the physically reasonable state range and the expected uncertainty level. These guidelines indicate that the parameters have interpretable roles and can be selected from noise statistics, physical constraints, and the expected outlier level rather than by exhaustive tuning.

For the consistency coefficient $\rho_{\alpha }$, its selection should depend on the reliability of the structural relation α=f(x). When the structural relation is accurately known and physically reliable, a smaller value of $\rho_{\alpha }$ is recommended because the filter should place more trust in the structural consistency correction. In this case, values in the range 0.2–0.4 can be used as an initial choice. In contrast, when the structural relation is uncertain, approximate, or affected by modeling errors, a larger value of $\rho_{\alpha }$ is preferable to avoid over-correcting the hidden variable toward a biased structural model. In such cases, values in the range 0.5–0.8 may be more appropriate. Therefore, $\rho_{\alpha }$ controls a practical trade-off between structural trust and measurement-updated hidden variable information.

**Figure 4:**
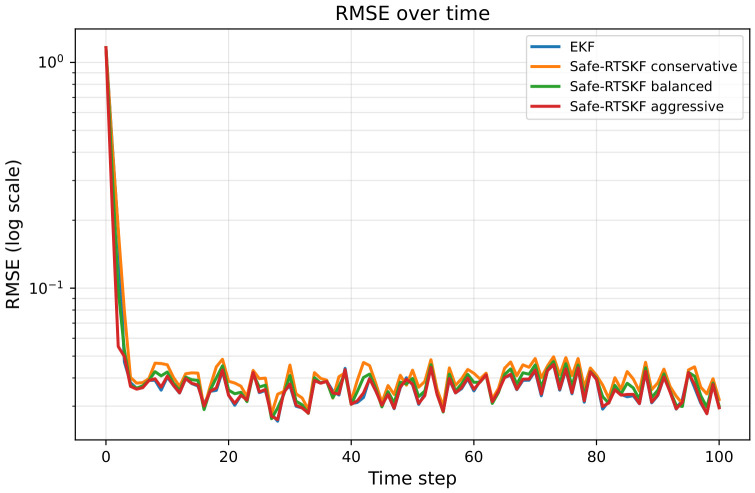
RMSE evolution over time under different safety parameter settings. The vertical axis is shown on a logarithmic scale to better visualize both the initial transient and the steady recursive behavior. The conservative, balanced, and aggressive settings correspond to different damping and consistency-correction strengths, illustrating the trade-off between robustness and tracking responsiveness.

Figure 4 illustrates the RMSE evolution over time and supports the observation that safety parameters affect the balance between robustness and tracking responsiveness.

### 3.5. Range–Bearing Target Tracking Under Outlier Measurements

To further evaluate the practical relevance of the proposed safety update mechanism in sensor systems, a range–bearing target tracking experiment was conducted. This experiment does not directly use the RTSKF hidden variable decomposition. Instead, it applies the safety update mechanism to EKF and constructs Safe-EKF. The purpose is to examine whether NIS gating, spectral-norm gain clipping, Joseph-form covariance updating, and covariance projection can improve recursive estimation robustness under outlier-contaminated sensor measurements.

The target state was defined as $x_{k}={\left[p_{x,k},p_{y,k},v_{x,k},v_{y,k}\right]}^{T}$. The trajectory length was set to T=80, and $N_{\mathrm{MC}}=100$ Monte Carlo runs were used. The initial true state was ${\left[1000,300,-8,4\right]}^{T}$, and the initial estimate was ${\left[1050,250,-6,2\right]}^{T}$. The process noise was generated with acceleration noise standard deviation $\sigma_{a}=0.15$. The range and bearing measurement noise standard deviations were $\sigma_{r}=5.0$ m and $\sigma_{\theta }=1^{\circ }$, respectively. Two scenarios were considered: a clean scenario with only Gaussian measurement noise and an outlier scenario in which 5% of the measurements were contaminated by abnormal perturbations. The outlier standard deviations were 50.0 m for range and $10^{\circ }$ for bearing. Bootstrap PF used 1500 particles.

The detailed parameter settings of this experiment are summarized in Table 12.

Table 13 reports the position RMSE, standard deviation, divergence rate, and runtime under the clean and outlier scenarios. In the clean scenario, EKF, Safe-EKF, UKF, and CKF achieved very similar position RMSE values. The RMSE of EKF was 7.1608, while that of Safe-EKF was 7.1680. This indicates that the safety update mechanism does not noticeably degrade estimation accuracy when the sensor measurements follow the nominal Gaussian noise assumption.

In the outlier scenario, Safe-EKF achieved the lowest position RMSE among the compared methods. The position RMSE of Safe-EKF was 9.1180, whereas the RMSE values of EKF, UKF, and CKF were 15.6053, 15.6225, and 15.6261, respectively. Compared with EKF, the relative RMSE reduction of Safe-EKF was

(102) $$\frac{15.6053-9.1180}{15.6053}\times 100\%\approx 41.57\%.$$

This improvement indicates that NIS gating and gain clipping effectively suppress abnormal innovation-driven updates. The Joseph-form covariance update and covariance projection further improve numerical stability during recursive filtering.

Table 14 reports the overall results by combining the clean and outlier scenarios. Safe-EKF obtained the lowest overall position RMSE, with an average value of 8.1430. Its runtime was higher than that of EKF but lower than those of UKF and CKF. Bootstrap PF required substantially more computation time, with an average runtime of 11.5692 s per Monte Carlo run.

To avoid relying only on mean RMSE, robust statistics were also computed. Table 15 shows the mean, median, IQR, and bootstrap 95% confidence interval. In the clean scenario, the median RMSE of Safe-EKF was almost identical to that of EKF, UKF, and CKF. In the outlier scenario, however, Safe-EKF achieved a median RMSE of 8.0355 and an IQR of 2.7466, both of which were substantially lower than those of EKF, UKF, and CKF. This indicates that Safe-EKF not only reduces the average error but also produces a more concentrated error distribution under abnormal measurements.

Paired significance tests were further conducted for the outlier scenario. As shown in Table 16, Safe-EKF significantly outperformed EKF, UKF, and CKF after Holm correction. The mean differences were all negative, indicating that Safe-EKF achieved lower RMSE than the corresponding baseline in paired Monte Carlo comparisons.

These results demonstrate that the proposed safety update mechanism is particularly useful when sensor measurements contain abnormal innovations. In normal Gaussian noise conditions, Safe-EKF behaves similarly to conventional EKF. Under outlier-contaminated measurements, it substantially improves tracking robustness while retaining a recursive Kalman-filter structure and moderate computational cost.

### 3.6. Coordinated Turn Benchmark

To further evaluate the safety update mechanism in a more challenging nonlinear sensor tracking scenario, a coordinated turn (CT) benchmark was constructed. Compared with the constant velocity range–bearing experiment, the CT benchmark involves nonlinear target motion, maneuvering dynamics, range–bearing measurements, and outlier-contaminated observations. This experiment is used to examine whether Safe-EKF can maintain robust recursive tracking performance under stronger motion nonlinearity.

The CT target state is defined as

(103) $$x_{k}={\left[p_{x,k},p_{y,k},v_{k},\psi_{k},\omega_{k}\right]}^{T},$$

where $p_{x,k}$ and $p_{y,k}$ are the target position components, $v_{k}$ is the speed, $\psi_{k}$ is the heading angle, and $\omega_{k}$ is the turn rate. The nonlinear coordinated turn motion model is

(104) $$x_{k+1}=f_{\mathrm{CT}}\left(x_{k}\right)+w_{k},$$

where

(105) $$f_{\mathrm{CT}}\left(x_{k}\right)=\left[\begin{array}{c} p_{x,k}+\frac{v_{k}}{\omega_{k}}\left[\sin\left(\psi_{k}+\omega_{k}\Delta t\right)-\sin\left(\psi_{k}\right)\right]\\ p_{y,k}+\frac{v_{k}}{\omega_{k}}\left[-\cos\left(\psi_{k}+\omega_{k}\Delta t\right)+\cos\left(\psi_{k}\right)\right]\\ v_{k}\\ \psi_{k}+\omega_{k}\Delta t\\ \omega_{k} \end{array}\right].$$

The sensor measurement is the standard range–bearing observation

(106) $$z_{k}=\left[\begin{array}{c} \sqrt{p_{x,k}^{2}+p_{y,k}^{2}}\\ \mathrm{atan}2(p_{y,k},p_{x,k}) \end{array}\right]+\varepsilon_{k}.$$

Three scenarios were considered: clean, outlier, and HighTurn. The clean scenario uses ordinary Gaussian process and measurement noise. The outlier scenario injects abnormal measurement perturbations into 5% of the sensor measurements. The HighTurn scenario increases the maneuvering difficulty by using a higher turn rate condition. The main CT benchmark parameters are summarized in Table 17.

Table 18 reports the scenario-wise results. In the clean scenario, EKF, Safe-EKF, UKF, and CKF achieved very similar RMSE values. The RMSE of Safe-EKF was 11.5694, which was almost the same as EKF, UKF, and CKF. This indicates that the safety update mechanism does not noticeably degrade estimation accuracy when the measurement noise follows the nominal Gaussian assumption.

In the outlier scenario, Safe-EKF achieved an RMSE of 17.9275, which was much lower than EKF, UKF, and CKF. Compared with EKF, the relative RMSE reduction was

(107) $$\frac{30.7530-17.9275}{30.7530}\times 100\%\approx 41.70\%.$$

This result confirms that the NIS gate and gain clipping mechanism can suppress abnormal innovation-driven corrections in nonlinear range–bearing tracking.

In the HighTurn scenario, Safe-EKF also achieved a lower RMSE than EKF, UKF, and CKF. Compared with EKF, the relative RMSE reduction was

(108) $$\frac{23.8081-14.9468}{23.8081}\times 100\%\approx 37.22\%.$$

This indicates that the safety update mechanism is also useful under stronger maneuvering dynamics, where nonlinear propagation and measurement updates become more challenging.

Table 19 summarizes the overall performance across the three CT scenarios. Safe-EKF achieved the lowest overall RMSE mean of 14.8146 and the smallest IQR of 3.5478. Its runtime was slightly higher than EKF but lower than UKF and CKF. Bootstrap PF required the largest computational cost and showed much larger error dispersion in this benchmark.

The overall RMSE reduction of Safe-EKF relative to EKF was

(109) $$\frac{22.0430-14.8146}{22.0430}\times 100\%\approx 32.79\%.$$

The runtime of Bootstrap PF was approximately

(110) $$\frac{9.1582}{0.0267}\approx 343.00$$

times that of Safe-EKF. These results show that Safe-EKF provides a favorable robustness–runtime trade-off in the CT benchmark.

**Figure 5:**
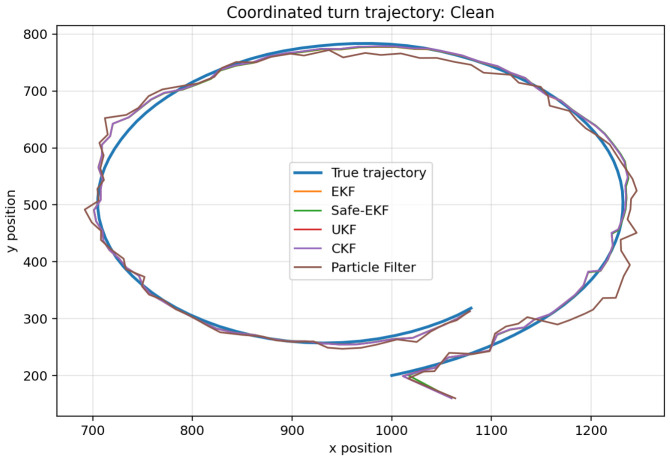
Coordinated turn trajectory tracking result in the clean scenario. Safe-EKF achieves a trajectory close to EKF, UKF, and CKF under nominal Gaussian measurement noise, indicating that the proposed safety update mechanism does not noticeably degrade tracking accuracy in normal conditions. Bootstrap PF is included as a sampling-based baseline.

**Figure 6:**
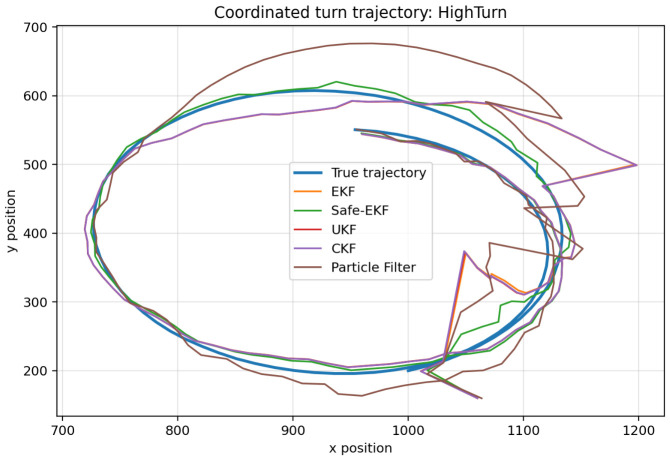
Coordinated turn trajectory tracking result in the HighTurn scenario. Safe-EKF maintains more stable tracking under stronger maneuvering dynamics, while conventional EKF-type baselines exhibit larger local deviations during the high-turn segment. Bootstrap PF is also shown as a sampling-based baseline and may deviate more in this representative run because of the prior proposal and finite particle approximation.

**Figure 7:**
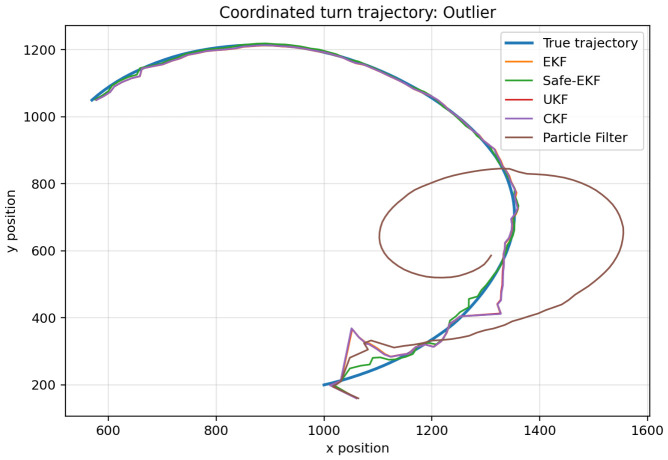
Coordinated turn trajectory tracking result in the outlier scenario. Safe-EKF suppresses outlier-induced trajectory drift more effectively than conventional EKF-type baselines by limiting abnormal innovation-driven updates through NIS gating and gain clipping. Bootstrap PF is more sensitive in this representative run because the implemented baseline uses prior proposal propagation and standard Gaussian likelihood weighting under outlier-contaminated measurements.

Figure 5, Figure 6 and Figure 7 show the trajectory tracking results under the clean, HighTurn, and outlier CT scenarios.

The CT benchmark further confirms the practical value of the safety update mechanism in nonlinear sensor tracking. In normal Gaussian noise conditions, Safe-EKF maintains accuracy similar to conventional EKF. Under outlier-contaminated measurements and high-turn-rate maneuvers, it achieves lower RMSE and more concentrated error distributions. This supports the use of safety-constrained recursive updates for robust real-time sensor state estimation.

### 3.7. Runtime and Computational Cost Analysis

Runtime is an important factor for sensor state estimation because many tracking, navigation, and monitoring systems require recursive online implementation. Therefore, this subsection further summarizes the computational cost of Safe-RTSKF, Safe-EKF, and the main baseline methods.

For Safe-RTSKF, the additional computational cost mainly comes from the two-stage hidden variable update, NIS gating, spectral-norm gain clipping, Joseph-form covariance updating, covariance spectral projection, and hidden variable consistency correction. Let *n* be the state dimension, *r* be the hidden variable dimension, and *m* be the sensor measurement dimension. The dominant matrix operations include covariance propagation, innovation covariance inversion, covariance updating, and eigenvalue-based spectral projection. Therefore, the approximate per-step computational complexity of Safe-RTSKF can be written as

(111) $$O(n^{3}+r^{3}+m^{3}),$$

when general dense matrix decomposition and inversion are used.

In contrast, the Bootstrap PF propagates and weights $N_{p}$ particles at each time step. Its approximate per-step computational complexity can be expressed as

(112) $$O(N_{p}C_{f}+N_{p}C_{h}+N_{p}),$$

where $C_{f}$ and $C_{h}$ denote the computational costs of state propagation and likelihood evaluation for a single particle, respectively. As the state dimension increases, a larger number of particles is usually required to maintain an adequate approximation of the posterior distribution. Therefore, the runtime of PF can increase rapidly in higher-dimensional or more nonlinear systems.

Table 20 summarizes the runtime results in the four-dimensional cyclically coupled system. Safe-RTSKF required 0.0552 s per Monte Carlo run, which was slightly higher than EKF, UKF, CKF, and Raw RTSKF, but still remained within the low-cost recursive filtering range. Bootstrap PF required 9.2302 s per Monte Carlo run, which was approximately 167 times slower than Safe-RTSKF:

(113) $$\frac{9.2302}{0.0552}\approx 167.21.$$

**Figure 8:**
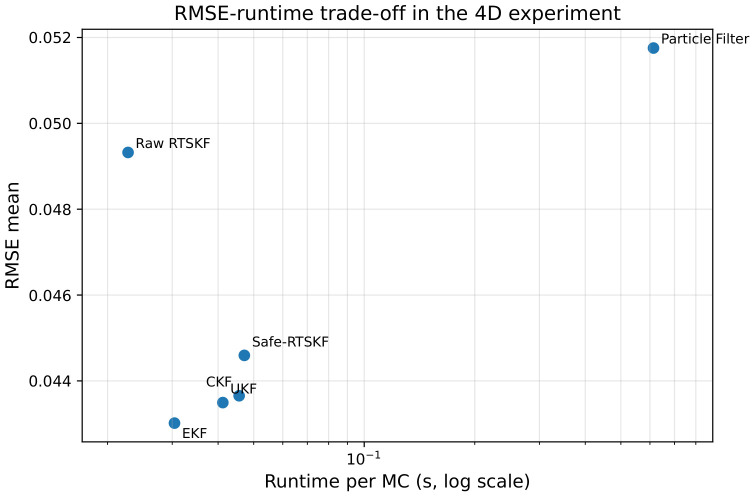
RMSE–runtime trade-off in the four-dimensional cyclically coupled system. Bootstrap particle filter achieves competitive RMSE but requires substantially higher computational time. Safe-RTSKF provides a better balance between estimation accuracy and recursive computational efficiency, making it more suitable for real-time filtering scenarios.

The RMSE–runtime relationship in Figure 8 further shows that Safe-RTSKF provides a practical trade-off between accuracy and computational efficiency.

The range–bearing tracking experiment further demonstrates the computational advantage of recursive Kalman-type filters over Bootstrap PF. As shown in Table 21, Safe-EKF required 0.0300 s per Monte Carlo run on average, whereas Bootstrap PF required 11.5692 s. Thus, Bootstrap PF was approximately 386 times slower than Safe-EKF:

(114) $$\frac{11.5692}{0.0300}\approx 385.64.$$

Although Safe-EKF was slower than standard EKF because of the additional safety operations, it remained faster than UKF and CKF in this experiment.

A similar trend was observed in the coordinated turn benchmark. Table 22 shows that Safe-EKF achieved the lowest overall RMSE with moderate runtime. Bootstrap PF required 9.1582 s per Monte Carlo run, whereas Safe-EKF required only 0.0267 s. Bootstrap PF was therefore approximately 344 times slower than Safe-EKF:

(115) $$\frac{9.1582}{0.0267}\approx 343.00.$$

The runtime results indicate that Safe-RTSKF and Safe-EKF introduce additional computation compared with their raw Kalman-type counterparts, but the increase is moderate. In contrast, Bootstrap PF requires substantially more computation time in the tested four-dimensional and target tracking scenarios. Therefore, the proposed safety-constrained recursive filtering framework is more suitable for sensor systems where robustness, numerical stability, and real-time implementation are all important. The results also suggest that the proposed method should be interpreted as a practical stability enhancement framework rather than a globally optimal nonlinear Bayesian estimator.

## 4. Discussion

The experimental results show that the proposed safety-constrained recursive filtering framework improves estimation stability mainly by addressing two sources of instability: unsafe innovation-driven updates and hidden variable inconsistency. In Raw RTSKF, the hidden variable is updated recursively and may gradually deviate from its structural relation with the state. Once this deviation enters the multiplicative state prediction model, it may produce biased predictions and further amplify subsequent update errors. The proposed hidden variable consistency correction directly targets this problem by pulling the updated hidden variable toward $f(\hat{x})$. Therefore, Safe-RTSKF is not merely a robustified Kalman update; it also introduces a structural correction mechanism that is specific to hidden variable two-stage filtering.

The ablation study provides a clearer interpretation of the roles of different modules. NIS gating, gain clipping, and damping alone were not sufficient to fully stabilize Raw RTSKF. Similar to many robust filtering ideas, these mechanisms can reduce the effect of abnormal innovations or excessive Kalman gains [9,10,15], but they cannot completely prevent covariance inflation and hidden variable drift. Covariance spectral projection was the key factor that eliminated numerical divergence, reducing the divergence rate to 0.00%. After this numerical stabilization was achieved, the hidden variable consistency correction further reduced the RMSE and substantially decreased the Monte Carlo variability. This suggests that covariance bounding mainly acts as a numerical safety mechanism, whereas consistency correction mainly improves estimation accuracy and repeatability.

The strongly nonlinear hidden variable experiments also indicate that Safe-RTSKF should be interpreted as a stability enhancement method rather than a universally optimal nonlinear filter. In the one-dimensional system, Bootstrap PF achieved the lowest RMSE because the state dimension was low and the particle approximation was effective. In the two-dimensional and four-dimensional systems, EKF, UKF, or CKF achieved slightly lower RMSE than Safe-RTSKF in some settings. Nevertheless, Safe-RTSKF consistently improved Raw RTSKF and achieved competitive performance with low-to-moderate runtime. This result is important because the objective of Safe-RTSKF is not to replace all nonlinear filtering methods, but to make the recursive two-stage hidden variable structure more stable under strong nonlinearity and high uncertainty.

The range–bearing and coordinated turn experiments further demonstrate the practical value of the safety update mechanism for sensor tracking problems. In the clean scenarios, Safe-EKF achieved almost the same accuracy as conventional EKF, UKF, and CKF, indicating that the safety constraints do not noticeably interfere with normal Gaussian noise updates. In the outlier scenarios, Safe-EKF substantially reduced the position RMSE and produced a more concentrated error distribution. This improvement can be explained by the NIS gate and gain clipping mechanism, which suppress abnormal innovation-driven corrections before they propagate into the recursive state estimate. In the HighTurn scenario of the coordinated turn benchmark, Safe-EKF also achieved lower RMSE than EKF, UKF, and CKF, suggesting that the safety update mechanism can improve robustness not only under outlier measurements but also under stronger maneuvering dynamics.

The comparison with Bootstrap PF should be interpreted carefully. Particle filtering is a general Bayesian approach and can be highly effective when a sufficient number of particles and suitable proposal or likelihood models are used [7,8]. In this paper, the PF baseline is implemented as a Bootstrap PF with prior proposal propagation, standard Gaussian likelihood weighting, and systematic resampling. Therefore, the results do not imply that Safe-RTSKF or Safe-EKF is superior to all particle filtering variants. Instead, the experiments show that, under the tested settings, the proposed safety-constrained recursive filters provide a better robustness–runtime trade-off than the Bootstrap PF baseline. The relatively weak performance of Bootstrap PF in the tracking experiments is mainly related to the use of the prior proposal and the standard Gaussian likelihood under maneuvering dynamics and outlier-contaminated measurements. These choices were kept simple to provide a transparent sampling-based baseline rather than an optimized PF implementation. More advanced PF variants, such as auxiliary particle filters, unscented particle filters, Rao–Blackwellized particle filters, and robust likelihood-based particle filters, should be considered in future comparative studies.

From the perspective of practical sensor systems, the proposed framework has several advantages. First, it preserves the recursive structure of Kalman-type filters and is therefore suitable for online implementation. Second, the safety modules are interpretable: NIS gating detects abnormal innovations, gain clipping limits excessive corrections, Joseph-form covariance updating improves covariance consistency, covariance projection prevents numerical collapse or explosion, and hidden variable consistency correction suppresses structural drift. Third, the framework can be adapted to different sensor estimation tasks. Safe-RTSKF is suitable for systems with explicit multiplicative hidden variable structures, whereas Safe-EKF demonstrates that the safety update principle can also be transferred to conventional nonlinear Kalman filtering for range–bearing tracking.

For ultra-fast sensor systems with very strict latency constraints, the proposed Safe-RTSKF/Safe-EKF framework should be applied selectively. If a nominal EKF is already sufficiently stable and the sensor measurements are clean, the additional safety operations may not be necessary. The proposed method is more suitable for sensor systems in which abnormal innovations, strong nonlinearity, hidden variable drift, or maneuvering dynamics can cause instability, and where a moderate increase in runtime is acceptable in exchange for improved robustness. Therefore, deployment in ultra-fast sensor systems is application-dependent and may require implementation-level optimization, such as fixed-size matrix operations, simplified covariance projection, or selective activation of safety modules only when abnormal innovations are detected.

However, several limitations remain. First, the current experiments are mainly simulation-based. The use of simulation is intentional in this study because it allows controlled and repeatable evaluation of strong nonlinearity, hidden variable coupling, initial uncertainty, outlier contamination, and runtime under identical Monte Carlo conditions. This controlled setting is necessary for isolating the effects of the proposed safety constraints and hidden variable consistency correction. Nevertheless, simulation cannot fully represent real sensor imperfections, such as calibration drift, asynchronous sampling, packet loss, measurement delay, and nonstationary noise statistics. Therefore, the current results should be interpreted as controlled evidence of algorithmic stability rather than final proof of field deployment performance. Future work should evaluate Safe-RTSKF and Safe-EKF on real radar, sonar, inertial navigation, robotic localization, industrial monitoring, or fault diagnosis datasets. Second, the current safety parameters, including NIS thresholds, gain clipping bounds, damping coefficients, covariance bounds, and consistency weights, are fixed. Although the sensitivity analysis shows that the method remains stable over a range of parameters, adaptive parameter selection would improve applicability to time-varying sensing environments. Third, the theoretical analysis provides local boundedness and consistency error contraction conditions rather than a global convergence guarantee. Stronger theoretical results under explicit observability, bounded noise, or stochastic stability assumptions remain an important future direction.

Another limitation is that the Safe-EKF tracking experiments verify the transferability of the safety update mechanism, but they are not direct applications of the full Safe-RTSKF hidden variable structure. This distinction is important. Safe-RTSKF is designed for nonlinear systems in which a hidden variable has a clear state-dependent structural definition. Safe-EKF, in contrast, is an application-oriented extension that applies the safety update mechanism to a standard EKF. Future work may investigate how to construct physically meaningful hidden variables for real sensor tracking or industrial sensing systems, so that the full Safe-RTSKF framework can be evaluated in more application-oriented scenarios.

Overall, the results suggest that combining safety-constrained recursive updates with structural consistency correction is a promising direction for robust nonlinear sensor state estimation. The proposed framework is especially useful when the system contains hidden variable coupling, abnormal sensor measurements, or high uncertainty, and when computational efficiency is important for online deployment.

## 5. Conclusions

This paper proposed Safe-RTSKF, a safety-constrained recursive two-stage Kalman filtering framework for robust sensor state estimation in strongly nonlinear systems with multiplicative hidden variable structures. The proposed method integrates NIS gating, spectral-norm Kalman gain clipping, update damping, Joseph-form covariance updating, covariance spectral projection, and hidden variable consistency correction into the recursive two-stage filtering process. The key motivation is to suppress two important instability sources in Raw RTSKF: unsafe innovation-driven updates and hidden variable drift from the structural relation α=f(x).

Local theoretical analysis was provided to explain the stability enhancement mechanism of Safe-RTSKF. The hidden variable consistency correction was shown to satisfy a local error bound and a contraction condition when the state estimation error is controlled. The NIS-gated and gain-clipped update was shown to have a bounded single-step correction, and the covariance spectral projection was shown to keep the state and hidden variable covariance matrices within prescribed bounds. These results do not constitute a global convergence proof, but they provide theoretical support for the local boundedness and consistency-improving behavior of the proposed method.

Monte Carlo experiments on one-dimensional, two-dimensional, and four-dimensional strongly nonlinear systems showed that Safe-RTSKF substantially improves Raw RTSKF. In particular, the ablation study demonstrated that covariance bounding is the key module for eliminating numerical divergence, while hidden variable consistency correction further reduces RMSE and Monte Carlo variability after numerical stability has been achieved. Parameter sensitivity analysis further indicated that the stability of Safe-RTSKF is not caused by a single accidental parameter setting.

The safety update mechanism was also transferred to the EKF framework to construct Safe-EKF for range–bearing and coordinated turn sensor tracking. In normal Gaussian noise scenarios, Safe-EKF maintained accuracy comparable to conventional EKF, UKF, and CKF. Under outlier-contaminated measurements, Safe-EKF significantly reduced tracking errors and produced more concentrated error distributions. In the coordinated turn benchmark, Safe-EKF also improved robustness under high-turn-rate maneuvering dynamics while maintaining moderate runtime. These results suggest that the proposed safety-constrained recursive update is useful for robust real-time sensor state estimation.

The proposed method should be interpreted as a practical stability enhancement framework rather than a globally optimal nonlinear Bayesian estimator. Bootstrap PF may achieve strong performance in low-dimensional nonlinear systems when sufficient particles and suitable likelihood models are used, but its computational cost can be much higher in higher-dimensional or target tracking scenarios. Safe-RTSKF and Safe-EKF provide a recursive Kalman-type alternative with interpretable safety mechanisms and favorable robustness–runtime trade-offs.

Future work will focus on three directions. First, Safe-RTSKF and Safe-EKF should be evaluated using real sensor datasets, such as radar tracking, sonar tracking, inertial navigation, robotic localization, industrial monitoring, and fault diagnosis data. Second, adaptive safety parameter selection should be investigated, including adaptive NIS thresholds, adaptive damping coefficients, adaptive covariance bounds, and adaptive hidden variable consistency weights. Third, the proposed safety-constrained recursive filtering idea can be extended to Safe-UKF, Safe-CKF, robust particle filtering variants, and unified sensor fusion frameworks for more complex nonlinear and non-Gaussian estimation problems.

## Figures and Tables

**Table 1:** Main notation used in the manuscript.

Symbol	Meaning
$x_{k}$	System state vector at time *k*.
$\alpha_{k}=f\left(x_{k}\right)$	State-dependent structural hidden variable. It is not an independent constant parameter.
$z_{k}$	Sensor measurement vector.
$w_{k}$	Process noise with covariance $Q_{k}$.
$\varepsilon_{k}$	Measurement noise with covariance $R_{k}$.
$v_{k}$	Target speed in the coordinated turn benchmark only.
$P_{k|k}^{x}$	Posterior state covariance.
$P_{k|k}^{\alpha }$	Posterior hidden variable covariance.
$Q_{k}^{\alpha }$	Design covariance describing the local uncertainty of the hidden variable prediction.
$R_{\mathrm{eff},k}^{\alpha }$	Effective measurement covariance for the hidden variable update at time *k*.

**Table 2:** Pseudocode of the proposed Safe-RTSKF algorithm.

**Input**	Initial estimates ${\hat{x}}_{0|0}$, ${\hat{\alpha }}_{0|0}$; covariances $P_{0|0}^{x}$, $P_{0|0}^{\alpha }$; measurements $\left\{z_{k}\right\}$; thresholds $\gamma_{\alpha }$, $\gamma_{x}$; clipping bounds $K_{\alpha ,\max}$, $K_{x,\max}$; projection bounds $P_{\min}$, $P_{x,\max}$, $P_{\alpha ,\max}$; damping coefficients $d_{\alpha }$, $d_{x}$; and consistency weight $\rho_{\alpha }$.
1	Predict the state using ${\hat{x}}_{k+1|k}=G\left({\hat{x}}_{k|k},{\hat{\alpha }}_{k|k}\right)$.
2	Propagate $P_{k+1|k}^{x}$ using Equation (17), including the multiplicative compensation term.
3	Form the local hidden variable prior ${\hat{\alpha }}_{k+1|k}$ and $P_{k+1|k}^{\alpha }$ using Equations (22)–(24).
4	Compute ${\hat{z}}_{k+1|k}^{\alpha }$, $\nu_{\alpha ,k}$, $R_{\mathrm{eff},k}^{\alpha }$, and $S_{\alpha ,k}$.
5	Apply NIS gating, spectral-norm gain clipping, and damping to obtain $K_{\alpha }^{\mathrm{safe}}$.
6	Update ${\hat{\alpha }}_{k+1|k+1}^{u}$ and $P_{k+1|k+1}^{\alpha ,u}$ using the Joseph form.
7	With ${\hat{\alpha }}_{k+1|k+1}^{u}$ fixed, compute the state innovation $\nu_{x,k}$ and apply the same safety update to obtain ${\hat{x}}_{k+1|k+1}^{u}$ and $P_{k+1|k+1}^{x,u}$.
8	Project $P_{k+1|k+1}^{x,u}$ and $P_{k+1|k+1}^{\alpha ,u}$ onto the prescribed spectral intervals.
9	Apply the structural consistency correction according to Equation (44).
10	Update $P_{k+1|k+1}^{\alpha }$ using Equation (45), followed by covariance projection.
**Output**	Posterior estimates ${\hat{x}}_{k+1|k+1}$ and ${\hat{\alpha }}_{k+1|k+1}$.

**Table 3:** Main settings of the compared filtering methods.

Method	Main Setting
EKF	Uses analytical or numerical Jacobians with the same initial state, initial covariance, process noise covariance, and measurement noise covariance as the other methods.
UKF	Uses the standard unscented transform with α=0.8, β=2, and κ=0.
CKF	Uses the standard third-degree spherical-radial cubature rule.
Robust UKF	Uses NIS–Huber innovation gating to suppress abnormal innovations.
Adaptive UKF	Updates the measurement noise covariance using an innovation-based adaptive rule with positive-definite clipping.
$H_{\infty }$-EKF	Uses a conservative robust update by adding an $H_{\infty }$-type regularization term to the innovation covariance.
Bootstrap PF	Uses prior proposal propagation, likelihood-based weight updating, and systematic resampling.
Raw RTSKF	Uses the recursive two-stage hidden variable update without safety constraints or consistency correction.
Safe-RTSKF	Uses NIS gating, spectral-norm gain clipping, update damping, Joseph-form covariance updating, covariance spectral projection, and hidden variable consistency correction.
Safe-EKF	Applies the safety update mechanism to the standard EKF for range–bearing and coordinated turn sensor tracking.

**Table 4:** Centralized reproducibility settings for the strongly nonlinear hidden variable experiments.

Setting	1D Strongly Nonlinear System	2D Coupled System	4D Cyclic Coupled System
State dimension	n=1	n=2	n=4
Hidden variable dimension	r=1	r=2	r=4
Trajectory length	T=100	T=100	T=100
Monte Carlo runs	$N_{\mathrm{MC}}=300$	$N_{\mathrm{MC}}=300$	$N_{\mathrm{MC}}=200$
Random seed	${\mathrm{seed}}_{i}=2025+i$	${\mathrm{seed}}_{i}=2025+i$	${\mathrm{seed}}_{i}=2025+i$
Divergence threshold	$1.0\times 10^{5}$	$1.0\times 10^{5}$	$1.0\times 10^{5}$
State transition	$x_{k+1}=x_{k}\sin\left(\beta x_{k}\right)+w_{k}$	$\begin{array}{c} x_{1,k+1}=0.8x_{1,k}\cos\left(\beta_{1}x_{2,k}\right)+w_{1,k},\\ x_{2,k+1}=0.8x_{2,k}\cos\left(\beta_{2}x_{1,k}\right)+w_{2,k} \end{array}$	$\begin{array}{cc} x_{i,k+1} & =0.8x_{i,k}\cos\left(\beta_{i}x_{i+1,k}\right)+w_{i,k},\\ & i=1,\ldots ,4,\;x_{5,k}=x_{1,k}. \end{array}$
Measurement function	$z_{k+1}=\sin\left(x_{k+1}\right)\cos\left(\sin\left(\beta x_{k+1}\right)\right)+\varepsilon_{k+1}$	$\begin{array}{c} z_{1,k+1}=\sin\left(x_{1,k+1}\right)\sin\left(\cos\left(\beta_{1}x_{2,k+1}\right)\right)+\varepsilon_{1,k+1},\\ z_{2,k+1}=\sin\left(x_{2,k+1}\right)\sin\left(\cos\left(\beta_{2}x_{1,k+1}\right)\right)+\varepsilon_{2,k+1} \end{array}$	$\begin{array}{cc} z_{i,k+1} & =\sin\left(x_{i,k+1}\right)\sin\left(\cos\left(\beta_{i}x_{i+1,k+1}\right)\right)+\varepsilon_{i,k+1},\\ & i=1,\ldots ,4,\;x_{5,k+1}=x_{1,k+1}. \end{array}$
Hidden variable	$\alpha_{k}=\sin\left(\beta x_{k}\right)$	$\alpha_{1,k}=\cos\left(\beta_{1}x_{2,k}\right),\;\alpha_{2,k}=\cos\left(\beta_{2}x_{1,k}\right)$	$\begin{array}{cc} \alpha_{i,k} & =\cos(\beta_{i}x_{i+1,k}),\\ & i=1,\ldots ,4,\;x_{5,k}=x_{1,k}. \end{array}$
Nonlinearity parameter	β=1.2	$\beta_{1}=1.2,\;\beta_{2}=1.1$	β=(0.9,1.0167,1.1333,1.25)
Process noise covariance	Q=0.01	$Q=0.01I_{2}$	$Q=0.01I_{4}$
Measurement noise covariance	R=0.05	$R=0.05I_{2}$	$R=0.05I_{4}$
Initial true state	$x_{0}=0.8+N\left(0,0.05^{2}\right)$	$x_{0}={\left[0.8,-0.6\right]}^{T}+N\left(0,0.05^{2}I_{2}\right)$	$x_{0}∼N\left(0,0.5^{2}I_{4}\right)$
Initial estimate	${\hat{x}}_{0|0}=0$	${\hat{x}}_{0|0}={\left[0,0\right]}^{T}$	${\hat{x}}_{0|0}={\left[0,0,0,0\right]}^{T}$
Initial state covariance	$P_{0|0}^{x}=2.0$	$P_{0|0}^{x}=I_{2}$	$P_{0|0}^{x}=I_{4}$
Initial hidden covariance	$P_{0|0}^{\alpha }=0.2$	$P_{0|0}^{\alpha }=0.2I_{2}$	$P_{0|0}^{\alpha }=0.2I_{4}$
Hidden process covariance	$Q^{\alpha }=10^{-3}$	$Q^{\alpha }=10^{-3}I_{2}$	$Q^{\alpha }=10^{-3}I_{4}$
NIS gate threshold	$\gamma_{x}=\gamma_{\alpha }=12.0$	$\gamma_{x}=\gamma_{\alpha }=16.0$	$\gamma_{x}=\gamma_{\alpha }=16.0$
Gain clipping bound	$K_{x,\max}=5.0,\;K_{\alpha ,\max}=1.20$	$K_{x,\max}=5.0,\;K_{\alpha ,\max}=1.20$	$K_{x,\max}=5.0,\;K_{\alpha ,\max}=1.20$
Update damping	$d_{x}=d_{\alpha }=0.75$	$d_{x}=d_{\alpha }=1.00$	$d_{x}=d_{\alpha }=1.00$
Consistency coefficient	$\rho_{\alpha }=0.55$	$\rho_{\alpha }=0.25$	$\rho_{\alpha }=0.25$
State covariance upper bound	$P_{\max}^{x}=3.0$	$P_{\max}^{x}=1.5$	$P_{\max}^{x}=1.5$
Hidden covariance upper bound	$P_{\max}^{\alpha }=0.30$	$P_{\max}^{\alpha }=0.20$	$P_{\max}^{\alpha }=0.20$
Particle number of Bootstrap PF	800	1200	1500
4D cyclic index convention	–	–	$x_{5,k}=x_{1,k}$ and $x_{5,k+1}=x_{1,k+1}$

**Table 5:** Comparison results on the one-dimensional strongly nonlinear system.

Method	RMSE	Div. Rate	Observation
Bootstrap PF	0.0479	0.00%	Lowest RMSE
Safe-RTSKF	0.0761	0.00%	Best non-PF method
CKF	0.0959	0.00%	Stable
EKF	0.1048	0.00%	Stable
$H_{\infty }$-EKF	0.1675	0.00%	Conservative
Adaptive UKF	418.7942	6.00%	Unstable
UKF	329.0286	8.00%	Unstable
Robust UKF	534.5966	8.00%	Unstable
Raw RTSKF	10,168.3269	89.00%	Severe divergence

**Table 6:** Comparison results on the two-dimensional coupled strongly nonlinear system.

Method	RMSE	Div. Rate	Observation
EKF	0.0577	0.00%	Lowest RMSE
UKF	0.0581	0.00%	Close to best
Robust UKF	0.0581	0.00%	Close to best
Bootstrap PF	0.0586	0.00%	Close to best
Safe-RTSKF	0.0589	0.00%	Improves Raw
Adaptive UKF	0.0605	0.00%	Slightly higher
CKF	0.0612	0.00%	Slightly higher
Raw RTSKF	0.0900	0.00%	Higher error
$H_{\infty }$-EKF	0.1728	0.00%	Conservative

**Table 7:** Additional comparison with augmented-state filtering baselines.

System	Method	RMSE Mean	RMSE Std.	Div. Rate	Runtime/s
1D	Augmented EKF	0.120767	0.016614	0.00%	0.019227
1D	Augmented UKF	0.120778	0.016617	0.00%	0.107360
1D	Raw RTSKF	0.106390	0.014151	0.00%	0.011523
1D	Safe-RTSKF	0.110389	0.016339	0.00%	0.012225
2D	Augmented EKF	0.179441	0.014347	0.00%	0.098707
2D	Augmented UKF	0.185390	0.016195	0.00%	0.157517
2D	Raw RTSKF	0.182539	0.014730	0.00%	0.084359
2D	Safe-RTSKF	0.179255	0.014339	0.00%	0.028414

**Table 8:** Comparison results on the four-dimensional cyclically coupled system.

Method	RMSE	Std.	Div. Rate	Time/s
EKF	0.0836	0.0072	0.00%	0.0274
UKF	0.0843	0.0069	0.00%	0.0435
CKF	0.0849	0.0068	0.00%	0.0401
Safe-RTSKF	0.0860	0.0093	0.00%	0.0552
Bootstrap PF	0.0900	0.0214	0.00%	9.2302
Raw RTSKF	0.1219	0.0426	0.00%	0.0385

**Table 9:** Ablation study of the proposed Safe-RTSKF modules.

Version	RMSE	RMSE Std.	MAE	Trace (*P*)	Div. Rate
M0: Raw RTSKF	10,327.1295	5805.0149	3081.6405	$8.2953\times 10^{11}$	93.00%
M1: +NIS Gate	10,325.3749	5773.5473	3076.7104	$8.2922\times 10^{11}$	93.33%
M2: +Gain Clip	10,357.6010	5775.7877	3092.1476	$1.5702\times 10^{11}$	93.00%
M3: +Damping	6483.4219	5255.9534	1833.8459	$3.0941\times 10^{9}$	88.33%
M4: +Cov. Bound	0.1853	0.3125	0.1295	0.0181	0.00%
M5: Full Safe-RTSKF	0.0758	0.0116	0.0569	0.0038	0.00%

**Table 10:** Summary of the one-factor parameter sensitivity analysis. The “Tested Values” column reports the complete candidate values used in each one-factor sweep.

Parameter	Tested Values	Fixed	Best	Observation
1D: $d_{x},d_{\alpha }$	0.55,0.65,0.75,0.85,0.95	0.75	0.95	Sensitive
1D: $\rho_{\alpha }$	0.35,0.45,0.55,0.65,0.75	0.55	0.35	Consistency helps
1D: $\gamma_{x},\gamma_{\alpha }$	6.0,9.0,12.0,15.0,18.0	12.0	18.0	Weak effect
1D: $P_{\alpha ,\max}$	0.10,0.20,0.30,0.50,0.80	0.30	0.10	Stable
1D: $P_{x,\max}$	1.5,2.0,3.0,5.0,8.0	3.0	1.5	Weak effect
2D: $d_{x},d_{\alpha }$	0.75,0.85,0.95,1.00	1.00	1.00	Fixed is best
2D: $\rho_{\alpha }$	0.15,0.25,0.35,0.45,0.55	0.25	0.25	Fixed is best
2D: $\gamma_{x},\gamma_{\alpha }$	10.0,12.0,16.0,20.0,24.0	16.0	12.0	Weak effect
2D: $P_{\alpha ,\max}$	0.10,0.20,0.30,0.50	0.20	0.20	Fixed is best
2D: $P_{x,\max}$	1.0,1.5,2.0,3.0	1.5	1.0	Stable

**Table 11:** RMSE variability statistics in the parameter sensitivity analysis.

Parameter	Mean	Range	CV	Stability
1D: damping	0.0778	0.0435	0.1707	Sensitive
1D: NIS gate	0.0761	0.0074	0.0281	High
1D: $P_{\alpha ,\max}$	0.0758	0.0057	0.0260	High
1D: $P_{x,\max}$	0.0758	0.0055	0.0259	High
1D: $\rho_{\alpha }$	0.0763	0.0146	0.0485	High
2D: damping	0.0620	0.0214	0.0961	Moderate
2D: NIS gate	0.0587	0.0140	0.0862	Moderate
2D: $P_{\alpha ,\max}$	0.0588	0.0153	0.0888	Moderate
2D: $P_{x,\max}$	0.0587	0.0140	0.0863	Moderate
2D: $\rho_{\alpha }$	0.0587	0.0143	0.0869	Moderate

**Table 12:** Parameter settings for the range–bearing target tracking experiment.

Parameter	Value
State vector	$x_{k}={\left[p_{x},p_{y},v_{x},v_{y}\right]}^{T}$
State dimension/measurement dimension	$n_{x}=4$, $n_{z}=2$
Sampling interval	Δt=1.0
Trajectory length	T=80
Monte Carlo runs	$N_{\mathrm{MC}}=100$
Initial true state	${\left[1000,300,-8,4\right]}^{T}$
Initial estimated state	${\left[1050,250,-6,2\right]}^{T}$
Initial covariance	$\mathrm{diag}(100^{2},100^{2},10^{2},10^{2})$
Acceleration noise standard deviation	$\sigma_{a}=0.15$
Measurement noise covariance	$R=\mathrm{diag}(5.0^{2},{\left(1^{\circ }\right)}^{2})$
Outlier ratio	5%
Outlier range standard deviation	50.0 m
Outlier bearing standard deviation	$10^{\circ }$
Safe-EKF NIS gate threshold	γ=9.21
Safe-EKF gain clipping upper bound	$K_{\max}=10^{4}$
Safe-EKF damping coefficient	d=1.0
Safe-EKF covariance upper bound	$P_{\max}=10^{6}$
Bootstrap PF particle number	$N_{p}=1500$

**Table 13:** Range–bearing target tracking results under the clean and outlier scenarios.

Scenario	Method	Position RMSE Mean	Position RMSE Std.	Divergence Rate	Runtime/s
Clean	EKF	7.1608	1.4548	0.00%	0.0087
Clean	Safe-EKF	7.1680	1.4654	0.00%	0.0167
Clean	UKF	7.1623	1.4457	0.00%	0.0257
Clean	CKF	7.1610	1.4418	0.00%	0.0241
Clean	Bootstrap PF	43.5160	58.1438	0.00%	6.4863
Outlier	EKF	15.6053	7.6745	0.00%	0.0234
Outlier	Safe-EKF	9.1180	4.1739	0.00%	0.0433
Outlier	UKF	15.6225	7.6455	0.00%	0.0664
Outlier	CKF	15.6261	7.6288	0.00%	0.0649
Outlier	Bootstrap PF	107.2322	162.5281	0.00%	16.6521

**Table 14:** Overall range–bearing target tracking results.

Method	Position RMSE Mean	Position RMSE Std.	Divergence Rate	Runtime/s
Safe-EKF	8.1430	3.2764	0.00%	0.0300
EKF	11.3831	6.9523	0.00%	0.0161
UKF	11.3924	6.9401	0.00%	0.0461
CKF	11.3936	6.9320	0.00%	0.0445
Bootstrap PF	75.3741	126.1467	0.00%	11.5692

**Table 15:** Robust RMSE statistics for the range–bearing target tracking experiment.

Scenario	Method	Mean	Median	IQR	95% CI
Clean	CKF	7.1610	7.0326	1.9588	[6.8880, 7.4452]
Clean	EKF	7.1608	6.9973	1.9964	[6.8772, 7.4614]
Clean	Bootstrap PF	43.5160	18.0179	37.3962	[32.8249, 55.9439]
Clean	Safe-EKF	7.1680	6.9995	1.9960	[6.8820, 7.4668]
Clean	UKF	7.1623	7.0314	1.9456	[6.8890, 7.4558]
Outlier	CKF	15.6261	14.0395	7.8418	[14.1791, 17.1571]
Outlier	EKF	15.6053	14.0259	7.3925	[14.1685, 17.2022]
Outlier	Bootstrap PF	107.2322	44.4456	111.1524	[78.6200, 142.4083]
Outlier	Safe-EKF	9.1180	8.0355	2.7466	[8.3764, 10.0025]
Outlier	UKF	15.6225	14.0334	7.8329	[14.2276, 17.2374]

**Table 16:** Paired significance tests between Safe-EKF and baseline methods under the outlier scenario.

Comparison	Mean Difference	Test	$p_{\mathrm{Holm}}$
Safe-EKF vs. EKF	−6.4873	Wilcoxon	$6.596\times 10^{-16}$
Safe-EKF vs. UKF	−6.5044	Wilcoxon	$6.596\times 10^{-16}$
Safe-EKF vs. CKF	−6.5080	Wilcoxon	$6.596\times 10^{-16}$

**Table 17:** Parameter settings of the coordinated turn benchmark.

Parameter	Value
Sampling interval Δt	1.0 s
Trajectory length *T*	100
Monte Carlo runs $N_{\mathrm{MC}}$	100
Position process noise standard deviation	0.05
Velocity process noise standard deviation	0.08
Heading noise standard deviation	$0.4^{\circ }$
Turn rate noise standard deviation	$0.15^{\circ }$
Range noise standard deviation	8.0 m
Bearing noise standard deviation	$1.2^{\circ }$
Outlier probability	5%
Outlier range standard deviation	80 m
Outlier bearing standard deviation	$12^{\circ }$
Bootstrap PF particle number	2000
NIS gate threshold	9.21

**Table 18:** Scenario-wise results of the coordinated turn benchmark.

Scenario	Method	RMSE Mean	RMSE Median	IQR	Divergence Rate	Runtime/s
Clean	EKF	11.5678	11.2684	2.5884	0.00%	0.0182
Clean	Safe-EKF	11.5694	11.2810	2.6364	0.00%	0.0235
Clean	UKF	11.5653	11.3790	2.8390	0.00%	0.0443
Clean	CKF	11.5720	11.3438	2.8703	0.00%	0.0429
Clean	Bootstrap PF	37.2148	21.0331	17.4660	0.00%	7.9350
Outlier	EKF	30.7530	24.3735	12.3837	0.00%	0.0244
Outlier	Safe-EKF	17.9275	12.1032	4.5793	0.00%	0.0310
Outlier	UKF	29.9553	24.2657	12.4180	0.00%	0.0585
Outlier	CKF	29.7866	24.2132	12.4174	0.00%	0.0546
Outlier	Bootstrap PF	234.4797	122.1791	320.5713	0.00%	10.7323
HighTurn	EKF	23.8081	21.3443	11.5101	0.00%	0.0205
HighTurn	Safe-EKF	14.9468	11.0852	3.0295	0.00%	0.0254
HighTurn	UKF	23.2833	21.5460	11.5796	0.00%	0.0488
HighTurn	CKF	23.8762	21.5465	11.5798	0.00%	0.0461
HighTurn	Bootstrap PF	110.7532	76.0934	143.0233	0.00%	8.8072

**Table 19:** Overall results of the coordinated turn benchmark.

Method	RMSE Mean	RMSE Median	IQR	Divergence Rate	Runtime/s
Safe-EKF	14.8146	11.4757	3.5478	0.00%	0.0267
UKF	21.6013	17.3303	13.3098	0.00%	0.0505
CKF	21.7449	17.3113	13.2272	0.00%	0.0479
EKF	22.0430	17.2112	13.6669	0.00%	0.0210
Bootstrap PF	127.4826	54.1934	136.3992	0.00%	9.1582

**Table 20:** Runtime comparison in the four-dimensional cyclically coupled system.

Method	Average Runtime/s	Total Runtime/s
EKF	0.0274	5.4830
Raw RTSKF	0.0385	7.6930
CKF	0.0401	8.0280
UKF	0.0435	8.6900
Safe-RTSKF	0.0552	11.0470
Bootstrap PF	9.2302	1846.0490

**Table 21:** Runtime comparison in the range–bearing target tracking experiment.

Method	Overall Position RMSE Mean	Average Runtime/s
Safe-EKF	8.1430	0.0300
EKF	11.3831	0.0161
UKF	11.3924	0.0461
CKF	11.3936	0.0445
Bootstrap PF	75.3741	11.5692

**Table 22:** Runtime comparison in the coordinated turn benchmark.

Method	Overall RMSE Mean	Average Runtime/s
Safe-EKF	14.8146	0.0267
EKF	22.0430	0.0210
UKF	21.6013	0.0505
CKF	21.7449	0.0479
Bootstrap PF	127.4826	9.1582

## Data Availability

The simulation code, random seed settings, raw Monte Carlo records, summary tables, statistical analysis results, and plotting scripts generated in this study will be made available from the corresponding author upon reasonable request.

## Abbreviations

The following abbreviations are used in this manuscript:

EKF	Extended Kalman Filter
UKF	Unscented Kalman Filter
CKF	Cubature Kalman Filter
PF	Particle Filter
RTSKF	Recursive Two-Stage Kalman Filter
Safe-RTSKF	Safety-Constrained Recursive Two-Stage Kalman Filter
NIS	Normalized Innovation Squared
RMSE	Root Mean Square Error
MAE	Mean Absolute Error
IQR	Interquartile Range
CT	Coordinated Turn

## References

[1] Kalman R.E. (1960). A new approach to linear filtering and prediction problems. J. Basic Eng..

[2] Kalman R.E., Bucy R.S. (1961). New results in linear filtering and prediction theory. J. Basic Eng..

[3] Bucy R.S., Senne K.D. (1971). Digital synthesis of non-linear filters. Automatica.

[4] Ito K., Xiong K. (2000). Gaussian filters for nonlinear filtering problems. IEEE Trans. Autom. Control.

[5] Julier S.J., Uhlmann J.K. (2004). Unscented filtering and nonlinear estimation. Proc. IEEE.

[6] Arasaratnam I., Haykin S. (2009). Cubature Kalman filters. IEEE Trans. Autom. Control.

[7] Gordon N.J., Salmond D.J., Smith A.F.M. (1993). Novel approach to nonlinear/non-Gaussian Bayesian state estimation. IEE Proc. F Radar Signal Process..

[8] Arulampalam M.S., Maskell S., Gordon N., Clapp T. (2002). A tutorial on particle filters for online nonlinear/non-Gaussian Bayesian tracking. IEEE Trans. Signal Process..

[9] Huber P.J. (1964). Robust estimation of a location parameter. Ann. Math. Stat..

[10] Masreliez C.J., Martin R.D. (1977). Robust Bayesian estimation for the linear model and robustifying the Kalman filter. IEEE Trans. Autom. Control.

[11] Mehra R.K. (1970). On the identification of variances and adaptive Kalman filtering. IEEE Trans. Autom. Control.

[12] Mehra R.K. (1972). Approaches to adaptive filtering. IEEE Trans. Autom. Control.

[13] Hassibi B., Sayed A.H., Kailath T. (1999). Indefinite-Quadratic Estimation and Control: A Unified Approach to H2 and H-Infinity Theories.

[14] Jia B., Xin M., Cheng Y. (2013). High-degree cubature Kalman filter. Automatica.

[15] He J., Sun C., Zhang B., Wang P. (2020). Maximum correntropy square-root cubature Kalman filter for non-Gaussian measurement noise. IEEE Access.

[16] Pagoti S.K., Vemuri S.I.D. (2022). Development and performance evaluation of correntropy Kalman filter for improved accuracy of GPS position estimation. Int. J. Intell. Netw..

[17] Zhao J., Netto M., Mili L. (2016). A robust iterated extended Kalman filter for power system dynamic state estimation. IEEE Trans. Power Syst..

[18] Friedland B. (1969). Treatment of bias in recursive filtering. IEEE Trans. Autom. Control.

[19] Alouani A.T., Xia P., Rice T.R., Blair W.D. (1993). On the optimality of two-stage state estimation in the presence of random bias. IEEE Trans. Autom. Control.

[20] Keller J.Y., Darouach M. (1997). Optimal two-stage Kalman filter in the presence of random bias. Automatica.

[21] Keller J.Y., Darouach M. (1999). Two-stage Kalman estimator with unknown exogenous inputs. Automatica.

[22] Haessig D.A., Friedland B. (1998). Separate-bias estimation with reduced-order Kalman filters. IEEE Trans. Autom. Control.

[23] Hsieh C.S., Chen F.C. (1999). Optimal solution of the two-stage Kalman estimator. IEEE Trans. Autom. Control.

[24] Li L.Q., Zhao D., Luo C.D. A novel interacting TS fuzzy multiple model by using UKF for maneuvering target tracking. Proceedings of the 2019 22nd International Conference on Information Fusion (FUSION).

[25] Zhou W., Hou J. (2019). A new adaptive high-order unscented Kalman filter for improving the accuracy and robustness of target tracking. IEEE Access.

[26] Allotta B., Caiti A., Chisci L., Costanzi R., Di Corato F., Fantacci C., Fenucci D., Meli E., Ridolfi A. (2016). An unscented Kalman filter based navigation algorithm for autonomous underwater vehicles. Mechatronics.

[27] Joerger M., Pervan B. (2013). Kalman filter-based integrity monitoring against sensor faults. J. Guid. Control Dyn..

[28] Tian Y., Huang Z., Tian J., Li X. (2022). State of charge estimation of lithium-ion batteries based on cubature Kalman filters with different matrix decomposition strategies. Energy.

[29] Li H., Sun H., Chen B., Shen H., Yang T., Wang Y., Jiang H., Chen L. (2022). A cubature Kalman filter for online state-of-charge estimation of lithium-ion battery using a gas–liquid dynamic model. J. Energy Storage.

[30] Cheng C., Wang W., Meng X., Shao H., Chen H.J. (2023). Sigma-mixed unscented Kalman filter-based fault detection for traction systems in high-speed trains. Chin. J. Electron..

[31] He D., Xu C., Zhu J., Du H. (2021). Moving horizon *H*_∞_ estimation of constrained multisensor systems with uncertainties and fading channels. IEEE Trans. Instrum. Meas..

[32] Wen C., Cheng X., Xu D., Wen C. (2017). Filter design based on characteristic functions for one class of multi-dimensional nonlinear non-Gaussian systems. Automatica.

[33] Wen C., Wang Z., Hu J., Liu Q., Alsaadi F.E. (2018). Recursive filtering for state-saturated systems with randomly occurring nonlinearities and missing measurements. Int. J. Robust Nonlinear Control.

[34] Sun X., He X., Wu X., Wen C. (2024). Internal and external double-cycle high-order Kalman filter design. IEEE Sens. J..

[35] Wen T., Jiang H., Cai B., Roberts C. (2024). High-speed train positioning using improved extended Kalman filter with 5G NR signals. IEEE Trans. Intell. Transp. Syst..

[36] Ahmed F., Conde M.H., Martínez P.L. (2023). Kalman filter-driven blind source localization for passive 3D ToF imaging. IEEE Sens. Lett..

[37] Liu W., Xu Y., Yin Z., Liu J., Gao R., Shi Z., Liang K., Huang Y. (2024). Robust error state FIR filter and its application in pedestrian tracking. IEEE Access.

[38] Rafique A.A., Al-Rasheed A., Ksibi A., Ayadi M., Jalal A., Alnowaiser K., Meshref H., Shorfuzzaman M., Gochoo M., Park J. (2023). Smart traffic monitoring through pyramid pooling vehicle detection and filter-based tracking on aerial images. IEEE Access.

